# Nodulating Legumes Are Distinguished by a Sensitivity to Cytokinin in the Root Cortex Leading to Pseudonodule Development

**DOI:** 10.3389/fpls.2018.01901

**Published:** 2019-01-08

**Authors:** Christopher Gauthier-Coles, Rosemary G. White, Ulrike Mathesius

**Affiliations:** ^1^Division of Plant Sciences, Research School of Biology, Australian National University, Canberra, ACT, Australia; ^2^CSIRO Agriculture, Canberra, ACT, Australia

**Keywords:** actinorhizal symbiosis, root cortex, cytokinin, evolution of nodulation, legumes, root growth

## Abstract

Root nodule symbiosis (RNS) is a feature confined to a single clade of plants, the Fabids. Among Fabids capable of RNS, legumes form root cortex-based nodules in symbioses with rhizobia, while actinorhizal species form lateral root-based nodules with actinomycetes. Cytokinin has previously been shown to be sufficient for “pseudonodule” initiation in model legumes. Here, we tested whether this response correlates with the ability to nodulate across a range of plant species. We analyzed the formation of pseudonodules in 17 nodulating and non-nodulating legume species, and 11 non-legumes, including nodulating actinorhizal species, using light and fluorescence microscopy. Cytokinin-induced pseudonodules arising from cortical cell divisions occurred in all nodulating legume species, but not in any of the other species, including non-nodulating legumes. Pseudonodule formation was dependent on the CRE1 cytokinin receptor in *Medicago truncatula*. Inhibition of root growth by cytokinin occurred across plant groups, indicating that pseudonodule development is the result of a specific cortical cytokinin response unique to nodulating legumes. Lack of a cortical cytokinin response from the *Arabidopsis thaliana* cytokinin reporter *TCSn::GFP* supported this hypothesis. Our results suggest that the ability to form cortical cell-derived nodules was gained in nodulating legumes, and likely lost in non-nodulating legumes, due to a specific root cortical response to cytokinin.

## Introduction

Root nodule symbiosis (RNS) is a key innovation allowing certain plants to associate with bacteria capable of converting atmospheric nitrogen into ammonia, a process known as nitrogen fixation (Sprent and Sprent, 1990). In exchange for this nitrogen, the plant provides the bacteria with a specialized habitat, a root-derived organ known as a nodule. The plant also provides the bacteria with a source of carbon in the form of sugars and organic acids (Udvardi and Poole, 2013). Due to the cost associated with this symbiotic relationship, RNS plants have evolved the ability to down-regulate nodule formation when sufficient nitrogen is present in the soil (Streeter, 1988). The ability to form RNS is likely dependent on a single predisposition event, the nature of which remains unknown, and is limited to a single clade, the Fabids (Soltis et al., 1995; Werner et al., 2014). This clade includes four orders, all of which contain species capable of RNS. The greatest diversity of RNS plants is found in the order Fabales (Sprent, 2009), which contains the legume family, Fabaceae. Within the Fabaceae the subfamily Papilionoideae contains the greatest number of RNS species and the majority of agriculturally significant species (Sprent, 2007). A characteristic phylogenetic trait of Papilionoids is a 50-kb inversion in their genome, which occurred early in their diversification, and this is theorized to have played a part in their rapid radiation, although the mechanisms for this are not clear (Cannon et al., 2014). Interestingly, a number of species within each legume subfamily are incapable of forming RNS, and phylogenetic studies have suggested that these species have likely lost this ability (Werner et al., 2014; Griesmann et al., 2018; van Velsen et al., 2018). An alternative hypothesis is that nodulation has evolved several times independently within the Fabid clade following the predisposition event (Soltis et al., 1995). The nature of the selection pressures as well as the specific genetic components that have led to this gain or loss of function remain unknown (Doyle, 2016).

Among RNS plants, nodule morphology is highly diverse. Nodulating legumes produce nodules originating predominately from the root cortex (e.g., Xiao et al., 2014). There is diversity in nodule morphology even among legumes, with some species forming “indeterminate nodules” which originate from the inner cortex, the layer of cells closest to the endodermis, and these nodules maintain an active meristem. Other legume species form “determinate nodules” and these originate from the outer and middle cortex and their growth terminates after nodule maturation (Hirsch, 1992). In the early stages of development, indeterminate as well as determinate nodules also involve some cell divisions originating from the pericycle (Hirsch, 1992). In contrast to legumes, other RNS plants produce nodules that originate predominantly from the root pericycle. These nodules are described as modified lateral roots. For example, plant species nodulating with filamentous actinorhizal bacteria (mainly *Frankia* spp.) form nodules that are initiated as lateral roots and later modified by *Frankia* to form nodules (Pawlowski and Sprent, 2007; Svistoonoff et al., 2014). These so-called “actinorhizal” symbioses are formed in some members of eight families of the Rosids, but not in legumes. It has been suggested that the cortical origin of legume nodules allowed nodulation “*sui generis*,” i.e., independent of lateral root formation (Hirsch and LaRue, 1997), but the nature of the predisposition for cortical-based nodules is not known.

Legume nodule development is a complex and tightly regulated process starting with the recognition between symbionts and the activation of rhizobia in the soil by root exudates, including flavonoids, followed by perception of Nod factors by the plant root by Nod factor receptors (Radutoiu et al., 2003). A signaling cascade is then initiated by the plant leading to nodule development; this involves the initiation of calcium spiking, followed by activation of a number of transcription factors including Nodule INception (NIN) and Nodulation Signaling Pathway1/2 (NSP1/2) (Oldroyd, 2013).

The term “Cytokinin” refers to a class of plant hormones ubiquitous across plants that are involved in many aspects of plant development (Werner and Schmülling, 2009; Hwang et al., 2012). In the root apical meristem, cytokinin inhibits meristematic activity by activating a repressor of auxin signaling, leading to changes in auxin transport proteins and subsequent auxin redistribution (Dello-Ioio et al., 2008; Rùžička et al., 2009). Similarly, in the pericycle, cytokinin inhibits lateral root emergence by acting on auxin transport proteins (Laplaze et al., 2007; Marhavý et al., 2014). In contrast to its inhibiting role on root growth, in nodulating legumes, cytokinin appears to play a role in activating meristematic activity in the normally non-meristematic cortex, which is necessary for nodule organogenesis (Gamas et al., 2017).

Cytokinin responses following infection by rhizobia are activated in the root cortex in both *M. truncatula* and *L. japonicus* (Plet et al., 2011; Held et al., 2014). Increased cytokinin concentrations have been measured in response to rhizobia in *M. truncatula* (van Zeijl et al., 2015) and *L. japonicus* (Reid et al., 2017) and the activation of genes involved in cytokinin synthesis are expressed in the cortex during nodulation in both species (Mortier et al., 2014; Reid et al., 2017). Additionally, using a cytokinin-responsive *TCSn::GUS* reporter line it was shown that the cortical cells of *M. truncatula* respond to exogenous cytokinin, suggesting that cytokinin signaling is active at the site of nodule organogenesis (Fonouni-Farde et al., 2017). CRE1 (CYTOKININ RESPONSE1), homologous to the Arabidopsis cytokinin receptor AHK4, is a membrane-bound cytokinin receptor necessary for nodulation in *M. truncatula* (Gonzalez-Rizzo et al., 2006; Plet et al., 2011). Cytokinin responses mediated by CRE1 during nodule organogenesis include the activation of *NIN* and *NSP2* expression (Plet et al., 2011; Ariel et al., 2012), as well as induction regulation of auxin transport (Plet et al., 2011; Suzaki et al., 2012), which is mediated by the induction of flavonoids, at least in *M. truncatula* (Ng et al., 2015).

Cytokinin is not only necessary but also sufficient for legume nodule initiation. An *L. japonicus* mutant expressing a constitutively active cytokinin receptor forms spontaneous nodules (Tirichine et al., 2007). Furthermore, exogenous application of cytokinin has been shown to lead to the formation of nodule-like, but uninfected, cortical cell-derived structures also known as “pseudonodules” in *M. sativa, L. japonicus, Trifolium repens*, and *Macroptilium atropurpureum* (Joshi et al., 1991; Relić et al., 1993; Hirsch et al., 1997; Mathesius et al., 2000a; Heckmann et al., 2011). In some cases, these pseudonodules expressed early nodulation genes and developed peripheral vasculature, suggesting that they closely resemble rhizobia-induced nodules (Hirsch et al., 1997; Fang and Hirsch, 1998; Mathesius et al., 2000a; Heckmann et al., 2011). There appears to be some variation in this response, with some ecotypes of *L. japonicus*, for example, showing little or no ability to form pseudonodules in response to exogenous cytokinin (Heckmann et al., 2011). Interestingly, cytokinin-induced pseudonodules have also been reported in the non-Fabid *Nicotiana tabacum* (Arora et al., 1959) and the non-legume actinorhizal RNS species *Alnus glutinosa* (Rodriguez-Barrueco and Bermudez De Castro, 1973). However, the role of cytokinin during actinorhizal nodulation is currently largely unknown.

Although these findings provide convincing evidence of the important role that cytokinin plays in cortical dedifferentiation and subsequent nodule development, to date, only a few nodulating legume species have been examined. Therefore, we investigated the actions of cytokinin across a broad phylogenetic and functional range of nodulating and non-nodulating legumes as well as non-legumes to find out whether the ability of plants to form pseudonodules in response to cytokinin correlates with their ability to nodulate with rhizobia or actinorhizal bacteria.

## Materials and Methods

### Species Selection

**FIGURE 1:**
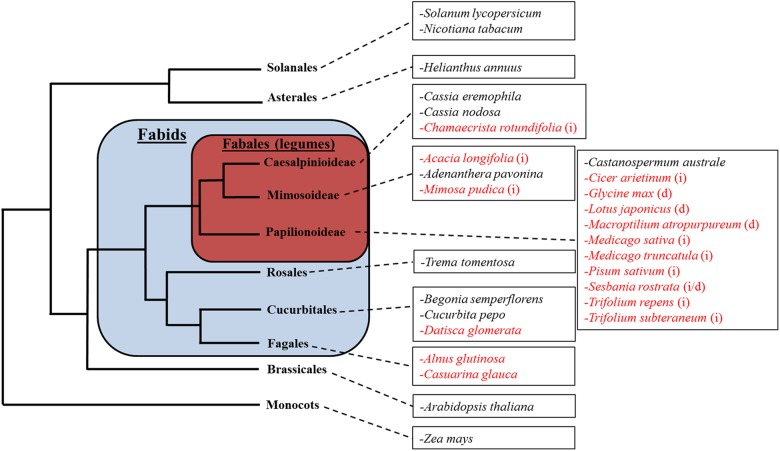
The relative phylogenetic relationship between subject species. Species in red indicate nodulating plant species. Legumes forming indeterminate nodules are marked with (i); legumes forming determinate nodules are marked with (d). Note that this tree does not reflect the exact phylogenetic relationship between the species shown, but merely illustrates their relative classification into different orders.

A total of 28 plant species were selected to represent the broad diversity of nodulating and non-nodulating Fabids as well as non-Fabids. These species can be placed into the following five categories: nodulating legumes (13 spp.); non-nodulating legumes (4 spp.); nodulating non-legume Fabids (3 spp.); non-nodulating non-legume Fabids (3 spp.); and non-Fabids (5 spp.) (Figure 1).

All species were propagated via seed germination. Seeds were acquired from Austrahort (Cleveland, QLD, Australia) for *Adenanthera pavonina, Castanospermum australe, M. atropurpureum, Mimosa pudica, Trema tomentosa*; Cleanseeds (Bungendore, NSW, Australia) for *Medicago sativa, T. repens, Trifolium subterraneum*; Royston Petrie Seeds (Mudgee, NSW, Australia) for *Acacia longifolia, Begonia semperflorens, Cassia eremophila, Cassia nodosa, Casuarina glauca, Glycine max, Solanum lycopersicum, Pisum sativum*; Mr Fothergill’s seeds (South Windsor, NSW, Australia) for *Cucurbita pepo, Helianthus annuus*, and *Zea mays*; Queensland Agricultural Seeds (Towoomba, QLD, Australia) for *Chamaecrista rotundifolia*; and the South Australian Research and Development Institute (Adelaide, SA, Australia) for *Medicago truncatula* cv. Jemalong A17. *Alnus glutinosa* seeds were harvested from a local park in Canberra. *Arabidopsis thaliana* Col-0 seeds were sourced from the Arabidopsis Biological Resource Centre (Ohio State University, United States). *Datisca glomerata* was supplied by Katharina Pawlowski (Stockholm University), *Sesbania rostrata* by Barry Rolfe (formerly Australian National University), *Cicer arietinum* by Angela Pattison (University of Sydney), *Lotus japonicus* by Brett Ferguson (University of Queensland), *N. tabacum* Wis. #381 by Spencer Whitney (Australian National University), the *cre1-1* mutant by Florian Frugier (Institute of Plant Sciences Paris-Saclay, France), and *A. thaliana TCSn::GFP* seeds by Bruno Müller (University of Zürich).

### Plant Propagation

Seeds were surface-sterilized in sodium hypochlorite solution 6% (w/v) [except 1% (w/v) for *A. thaliana, B. semperflorens, D. glomerata*, and *N. tabacum*]. Some species required pre-germination seed treatments (see Supplementary Table 1 for corresponding treatments per species) which included (1) acid treatment: soaking in 2 M sulfuric acid for 30 s prior to sterilization (except for *A. pavonina* and *C. nodosa*, which were soaked for 45 min); (2) stratification: dark storage at 4°C for 48 h after sterilization; (3) heat treatment: soaking in freshly boiled water for 1 h prior to sterilization; (4) soaking: soaking in water at 21°C overnight prior to sterilization. Most species were grown on sterile agar plates (16 h day, 8 h night with a light intensity of approximately 120 μmole photons m^-2^ s^-1^; 23°C). Plates contained Fåhraeus medium (Fåhraeus, 1957) for most plants, except for *D. glomerata*, which was grown on Hoagland’s nutrient agar (Hoagland and Arnon, 1950) (Supplementary Table 1); media was free of nitrogen with the exception of very small-seeded species which were supplemented with 0.5 mM KNO_3_ (Supplementary Table 1). Larger-seeded species were grown in pots (600 mL volume) containing autoclaved grade 3 vermiculite and kept in a glasshouse at 15/25°C (min/max for night/day) and supplemented with liquid N-free Fåhraeus medium once a week. To determine the effect of nitrogen on pseudonodule formation, 10 mM KNO_3_ was added to their respective media where indicated.

### Rhizobia Inoculation

*Mesorhizobium ciceri* strain CC119*2, Mesorhizobium loti* strain MAFF303099, *Sinorhizobium meliloti* strain 1021, and *Sinorhizobium fredii* NGR234 strain ANU280 were cultured on Bergersen’s Modified Medium (BMM) (Gresshoff et al., 1977) agar plates for 48 h at 28°C. Before inoculation, single colonies of rhizobia were grown overnight in liquid BMM on a shaking incubator at 28°C. Seeds of *L. japonicus, C. arietinum, M. atropurpureum, M. sativa*, and *M. truncatula* were germinated as described above and inoculated either when the main root was approximately 5 cm long, or in the case of *C. arietinum*, which was grown in pots, when seedlings were 5 days old. Plants grown on plates were inoculated either by flooding treatment, whereby seedlings were immersed in an aqueous solution containing liquid rhizobia culture for 60 s, which was then decanted, or in the case of *C. arietinum* by pipetting 1 mL of culture onto the soil surface. The optical density at 600 nm (OD600) of each culture was adjusted to 0.05, or 0.1 in the case of *M. ciceri*, just before inoculation.

To confirm that pseudonodules contained no rhizobia, 10 individual *C. arietinum* pseudonodules from different plants were surface-sterilized in aqueous sodium hypochlorite solution 1% (w/v) for 5 min and then thoroughly rinsed with sterile water. Pseudonodules were then crushed in an Eppendorf tube using a sterile glass rod and the resulting mixture was cultured on BMM agar plates for 48 h at 28°C. Single colonies of bacteria were grown overnight in liquid BMM on a shaking incubator at 28°C and re-inoculated onto untreated *C. arietinum* plants to test whether they induced nodules, or whether they were other contaminants. *C. arietinum* pseudonodules were chosen as they most closely resembled rhizobia-induced nodules. In addition, solvent-treated plants of each species were included to ensure that nodules did not result from rhizobial contamination in the glasshouse.

### Cytokinin Treatments

Five different types of cytokinins were used in this study: the synthetic cytokinin BAP (benzylaminopurine) as well as four endogenous cytokinins, *cis*-zeatin, *trans*-zeatin, dihydro-zeatin, and kinetin (all form Sigma Chemicals). For plate-grown plants, cytokinin was administered once via flooding, whereby seedlings were immersed in 20 mL of cytokinin solution for 20 s on the plate before decanting the solution. Cytokinin was applied to plate-grown plants once the main root measured more than 3 cm. For species grown in vermiculite, plants were watered with 50 mL cytokinin solution 1 week after germination and a second time 1 week later. Cytokinin concentrations varied across experiments from 10 nM to 40 μM. We initially treated all plant species with 1 and 10 μM BAP, but if no obvious signs of root growth inhibition were observed at those concentrations, we varied the concentrations of BAP to concentrations that resulted in visible changes in root growth. In nodulating legumes, we also tested additional concentrations of BAP to gain a more detailed picture of active concentrations. Therefore, not all species were treated with the same BAP concentrations in the presented experiments. Stock solutions of 10 mM of the various cytokinins were made in dimethyl sulfoxide (DMSO, Sigma Chemicals) and treatment solutions were made up with sterile distilled water, all solutions were filter sterilized. Appropriately diluted and filter-sterilized DMSO was used as a negative control treatment.

### Quantifying Pseudonodules

Plate-grown plants were harvested 4 weeks following treatments; plants grown in vermiculite were harvested 3 weeks after the first treatment and gently rinsed in tap water. Pseudonodules were counted with the aid of a magnifying glass or stereo microscope. Pseudonodules were identified by their bulbous shape, which contrasted with emerging lateral roots identified by their clearly recognizable conical shape.

A Chi-square analysis was performed between each cytokinin treatment and control to determine the statistical significance of the number of replicates producing pseudonodules using Prism from GraphPad (La Jolla, CA, United States). A non-parametric Kruskal–Wallis test with a Dunn *post hoc* test was performed between treatments producing pseudonodules in order to determine cytokinin concentration-dependent frequency of pseudonodulation.

### Root Growth Assays

Following harvesting and nodule counting, plants grown in agar, as well as some grown in vermiculite were scanned on a flat-bed scanner (Epson Perfection V700 Photo). A ruler was scanned next to the roots for calibration. Main root length, lateral root length, and total root numbers were determined using ImageJ^1^. For vermiculite-grown plants with highly complex root architecture, fresh root weight was measured as a proxy for the overall length of the root system. Analysis of variance (ANOVA) with Tukey *post hoc* test was performed between treatments for each species using Prism.

### Anatomical Analysis of Roots

Transverse cross-sections of cytokinin-treated roots and control roots were examined in all species. At least five 1 cm-long root segments from different plants were serially cross-sectioned per species and treatment. This was done in order to qualitatively identify the cellular origin of pseudonodules. Small root segments were embedded in 3% (w/v) DNA-grade agarose (Amresco). Transverse cross-sections of 100 μm were made using a vibratome (1000plus, Vibratome Company). Cross-sections were transferred to glass slides and visualized using a compound microscope (Leica DM5500B) using brightfield or fluorescence illumination (excitation 360 ± 40 nm, emission 470 ± 40 nm) with a Leica DFC7000T color digital camera. Fluorescence microscopy allowed clear identification of the endodermis to assess any cell division in the cortex. To ensure that pseudonodules contained no infected cells, *C. arietinum* pseudonodules and rhizobia-infected nodules were sectioned and stained with Toluidine Blue (0.05%, pH 4.4) to identify infected nodules (Maunoury et al., 2010).

### Assessing Cytokinin Response in *A. thaliana TCSn::GFP* Reporter Line Using Confocal Microscopy

Following sterilization and cold-treatment, *A. thaliana TCSn::GFP* seeds (Zürcher et al., 2013) were grown on the surface of glass slides coated with a thin film of N-free Fåhraeus agar, on which roots could be directly visualized through a microscope. These slides were placed in sterile magenta jars containing a layer of dental wax on the base to secure the slides. The magenta jars were placed in a temperature-controlled growth chamber at 20°C, with a 16 h light period at 120 μmole photons m^-2^ s^-1^ light intensity. After germination, seedlings were grown for four more days.

Prior to BAP treatment, the GFP fluorescence in roots of five seedlings was recorded by imaging 1 mm from the main root tip using a Leica SP8 laser scanning confocal microscope (488 nm excitation, 500–550 nm emission) with corresponding transmitted brightfield image. In order to obtain a vertical profile of fluorescence through each root, a series of optical sections was collected using the confocal Z-stack software.

Seedlings were then incubated in a 1 μM BAP solution for 20 h in the dark, as described by Zürcher et al. (2013). Following cytokinin incubation, the fluorescence from the same part of each root was recorded using the same confocal imaging settings. Pre-cytokinin and post-cytokinin images were then compared using the Leica LasX image analysis software in order to quantify the GFP signal in separate cell layers. To statistically compare the change in fluorescence, the pre-cytokinin and post-cytokinin signal intensity values were analyzed using a two-tailed Student’s *t*-test for each cell layer using Prism.

*TCSn::GFP* expression in whole seedling roots was visualized using a fluorescence stereomicroscope (Leica M205FA) after excitation with a ET Blue LP filter system (excitation 470 nm, emission 515 nm long pass filter). Photos were taken with a Leica DFC550 digital camera.

## Results

### Morphological and Anatomical Effects of Cytokinin on Root Development

**FIGURE 2:**
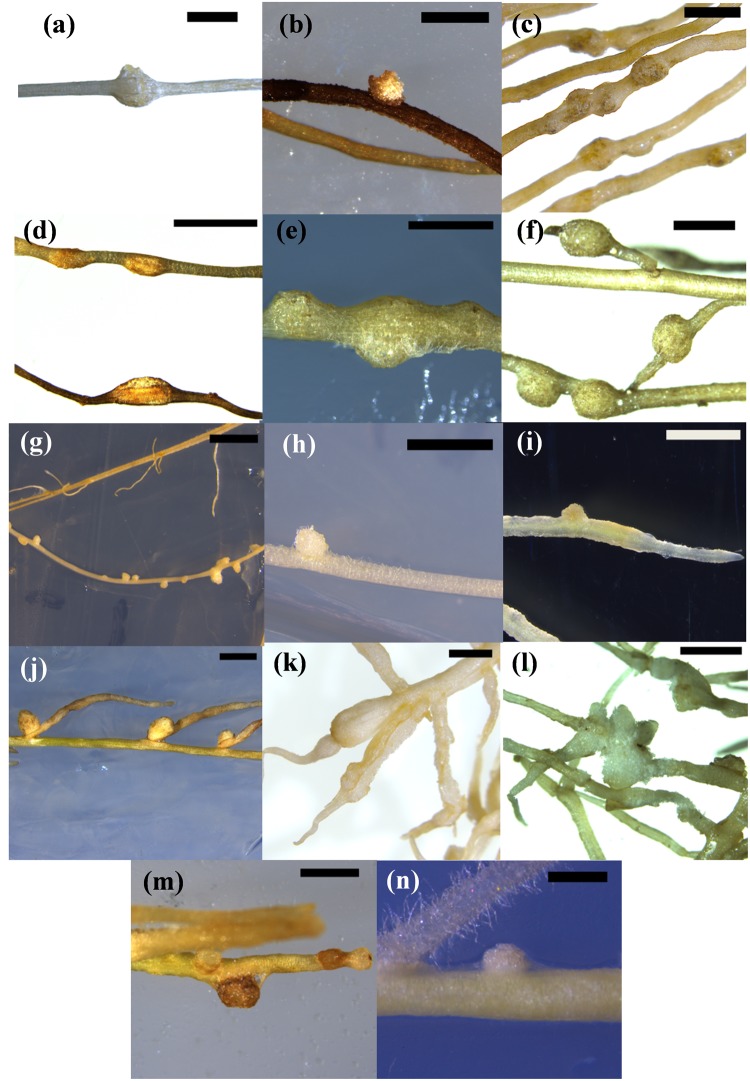
*Pseudonodules in nodulating legume species*. **(a)** *Acacia longifolia* 5 μM BAP; **(b)** *Chamaecrista rotundifolia* 10 μM BAP; **(c)** *Cicer arietinum* 10 μM BAP; **(d)** *Glycine max* 1 μM BAP; **(e)** *Lotus japonicus* 1 μM BAP; **(f)** *Macroptilium atropurpureum* 10 μM BAP; **(g)** *Medicago sativa* 10 nM BAP; **(h)** *Medicago sativa* untreated spontaneous nodule; **(i)** *Medicago truncatula* 1 μM BAP; **(j)** *Mimosa pudica* 10 μM BAP; **(k)** *Pisum sativum* 10 μM BAP; **(l)** *Sesbania rostrata* 1 μM BAP, **(m)** *Trifolium repens* 10 μM BAP; **(n)** *Trifolium subterraneum* 10 μM BAP. Bars **(a,c,d,g,j,k)** 2 mm; **(b,e,f,h,i,l,m,n)** 1 mm.

The synthetic cytokinin BAP has been used previously to induce pseudonodules in legumes (e.g., Mathesius et al., 2000a; Heckmann et al., 2011) and was therefore used to screen all 28 species. BAP-induced nodule-like structures (“pseudonodules”) formed on roots of all nodulating legume species examined, with some morphological variation between species (Figure 2). The ability to form pseudonodules did not seem to be more frequent or efficient in legumes forming either determinate or indeterminate nodules (as indicated in Figure 1), but varied even within a genus (see below). Across species, pseudonodules were fully developed between 2 and 4 weeks post-BAP treatment and formed in species grown in plates as well as in pots containing vermiculite. *Mimosa pudica* was grown in both plates and vermiculite and both growth media resulted in pseudonodulation after BAP treatment. No pseudonodules were observed in any of the non-nodulating legumes, or in any of the non-legume species, irrespective of their growth on plates or in vermiculite (Supplementary Table 2). No pseudonodules or cortical cell divisions were seen after application of 1 μM BAP to roots of the Medicago *cre-1* mutant (0/20 roots), which is defective in the cytokinin receptor gene *CRE-1* (Supplementary Figure 1e), compared to successful pseudonodules found in wild-type roots (9/20 roots). None of the solvent-treated plants showed any nodulation except the legume *M. sativa*, which showed some degree of spontaneous nodulation as previously reported (Figure 2h; Joshi et al., 1991).

**FIGURE 3:**
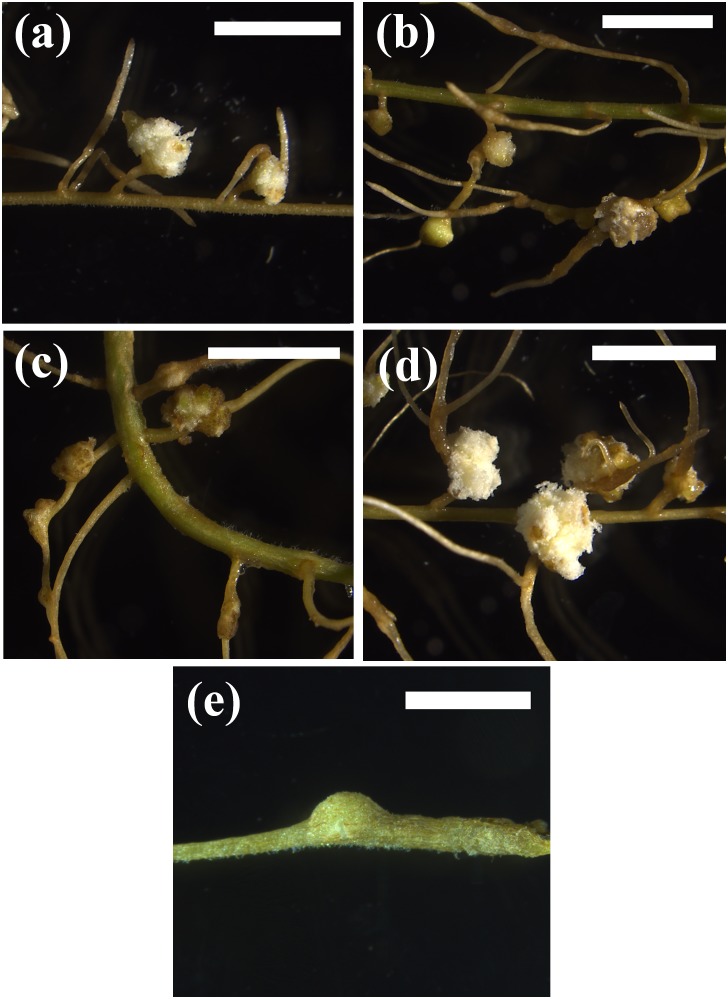
Phenotypic response of different cytokinins on *M. atropurpureum*. **(a)** 10 μM BAP. **(b)** 50 μM *cis*-zeatin. **(c)** 10 μM dihydrozeatin. **(d)** 50 μM kinetin. **(e)** 60 μM *trans*-zeatin. Pseudonodules are shown 4 weeks after treatment. Bars **(a–d)** 500 μm; **(e)** 200 mm.

We also tested whether endogenous cytokinins were able to induce pseudonodules. For this experiment, we chose one legume species that readily formed pseudonodules with BAP, *M. atropurpureum*, and two non-legumes. For the non-legumes, we chose *A. glutinosa* and *N. tabacum*, because in these species pseudonodules had been reported in previous studies (Arora et al., 1959; Rodriguez-Barrueco and Bermudez De Castro, 1973). In addition, *A. glutinosa* represented a nodulating actinorhizal species and *N. tabacum* represented a non-nodulating non-fabid. These hormones were tested at concentrations of 10, 20, 50, 60, and 100 μM. Application of *cis*-zeatin, *trans*-zeatin, dihydro-zeatin, or kinetin all induced pseudonodules in the nodulating legume *M. atropurpureum* (Figure 3), showing that all applied cytokinins could induce pseudonodules on a nodulating legume, although with some differences in resulting morphology and effective concentrations. The photos in Figure 3 show the lowest tested cytokinin concentrations that led to the formation of pseudonodules, although pseudonodules were also formed at higher concentrations. However, no pseudonodules were found after application of these endogenous cytokinins at any of the concentrations in the actinorhizal species *A. glutinosa* or the non-fabid *N. tabacum*, although some cortical cell-swelling was observed in *A. glutinosa* (Supplementary Figure 1).

Across nodulating legume species, pseudonodules emerged from both main roots and lateral roots. A notable exception was *M. pudica*, where pseudonodules emerged exclusively from the junction between the main root and lateral roots (Figure 2j). In *P. sativum*, significant root swelling due to expanded cortical cells (Figure 2k) resulting from BAP treatment made it difficult to enumerate pseudonodules without sectioning the root. However, cross sections clearly showed the formation of pseudonodule primordia (Supplementary Figure 2).

The frequency of pseudonodulation was species- and concentration-dependent, but there was no interspecific trend in the frequency of pseudonodulation as there was significant variation between species in the optimum BAP concentration. In some species (*M. truncatula* and *L. japonicus*), higher BAP concentrations lead to lower pseudonodulation frequency (Supplementary Table 2) possibly due to the concomitant inhibition of root growth by BAP.

**FIGURE 4:**
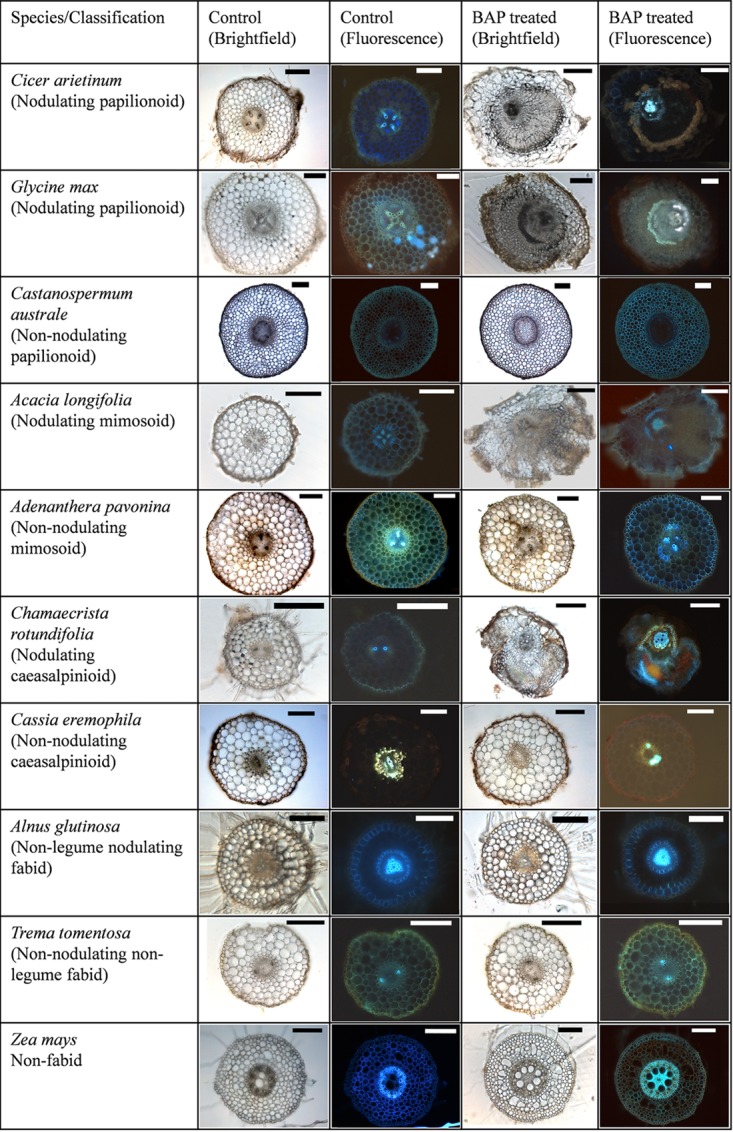
Cytokinin-induced organogenesis is a conserved feature of nodulating legumes. A range of examples of nodulating and non-nodulating legumes from the three legume subfamilies are shown along with actinorhizal plants, non-nodulating Fabids, and non-nodulating non-Fabids. *Cicer arietinum* 1 μM BAP; *Glycine max* 10 μM BAP; *Castanospermum australe* 10 μM BAP; *A. longifolia* 10 μM BAP; *Adenanthera pavonina* 20 μM BAP; *C. rotundifolia* 10 μM BAP; *Cassia eremophila* 20 μM; *Alnus glutinosa* 10 μM BAP; *Trema tomentosa* 20 μM BAP; *Zea mays* 20 μM BAP. All bars = 200 μm, except BAP-treated *C. arietinum, A. longifolia* (500 μm), and control and treated *A. glutinosa* (100 μm).

Cross sections through BAP-treated roots revealed that pseudonodules (and spontaneous nodules of *M. sativa*) appeared to originate from cell divisions in the root cortex (outside the autofluorescent endodermis) (Figure 4 and Supplementary Figure 2). In certain species, however, such as *A. longifolia* (Figure 4) the endodermis was no longer clearly discernible, making it difficult to identify the cellular origins of the structure.

**FIGURE 5:**
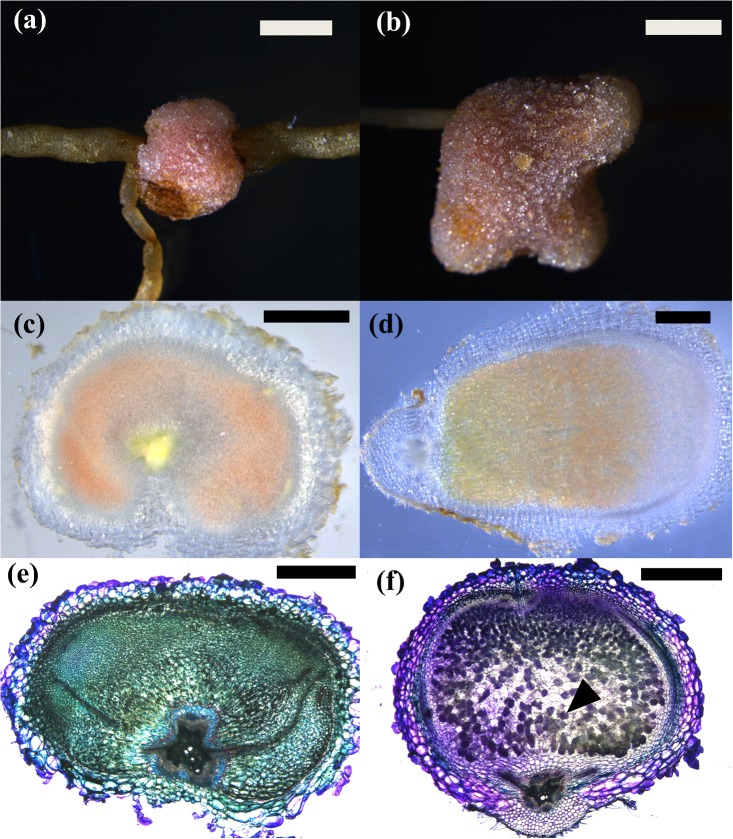
Phenotypes of 4-week-old pseudonodules and infected nodules in *C. arietinum*. **(a,c,e)** *C. arietinum* treated with 5 μM BAP; **(b,d,f)** *C. arietinum* infected with *Mesorhizobium ciceri.* **(a,b)** Stereo microscope image of mature (pseudo)nodules showing pink coloration from the outside. Bar = 500 μm. **(c,d)** Cross sections through mature (pseudono)nodules. While pseudonodules form in a wide area around the root, infected nodules are more elongated. **(e,f)** Toluidine Blue-stained cross sections of (pseudo)nodules showing dark purple, large infected cells in infected nodules (black arrow) **(f)** and smaller cells without intracellular staining inside the pseudonodule, suggesting that these cells are not infected **(e)**.

In *C. arietinum*, pseudonodules consistently contained a pink pigment in the center, which made them appear similar to leghemoglobin-containing rhizobia-induced nodules (Figure 5). Control-treated plants showed no pink pigmentation. To test whether these pink pseudonodules contained rhizobia or not, bacteria isolated from surface-sterilized pseudonodules were re-inoculated onto sterile-grown chickpea plants. None of these plants developed nodules. In addition, sections of rhizobia-induced nodules displayed characteristic staining indicative of infected nodule cells after Toluidine Blue staining, whereas pseudonodules did not appear to contain any infected cells (Figure 5).

Non-nodulating legumes, nodulating non-legume Fabids, non-nodulating non-legume Fabids, and non-Fabids, none of which developed visible pseudonodules, also showed no cortical cell divisions in response to BAP, confirmed in serial sections of several roots for each species (Figure 4 and Supplementary Figures 2, 3). One exception was that in the non-Fabid *H. annuus* (sunflower), BAP induced limited cortical cell division; however, this never advanced to the stage of organogenesis (Supplementary Figure 1d). Cell swelling, mainly of cortical cells, was another cytokinin-induced phenotype observed across several plant species irrespective of their ability to nodulate or form pseudonodules (Supplementary Figure 1). However, cell swelling was also observed to some extent in the *cre1* mutant of *M. truncatula* (Supplementary Figure 1e), and is therefore likely an unrelated effect.

To test if the ability of BAP to form pseudonodules was correlated with the species’ ability to respond to BAP with other known cytokinin-induced root phenotypes, we determined root length and lateral root numbers; in pot-grown species with complex root systems, root weight was measured as a proxy for root length and root numbers. Both assays revealed a consistent and usually dose-dependent reduction in either root length or lateral root number or both, following BAP treatment, irrespective of a species’ ability to form pseudonodules (Supplementary Figures 4, 5), except for *S. lycopersicum, C. nodosa*, and *A. pavonina*, which did not respond to any applied BAP concentration. This suggests that pseudonodulation is the result of a specific response to BAP in nodulating legumes.

### Comparison of BAP-Induced Pseudonodules and Infected Nodules

**FIGURE 6:**
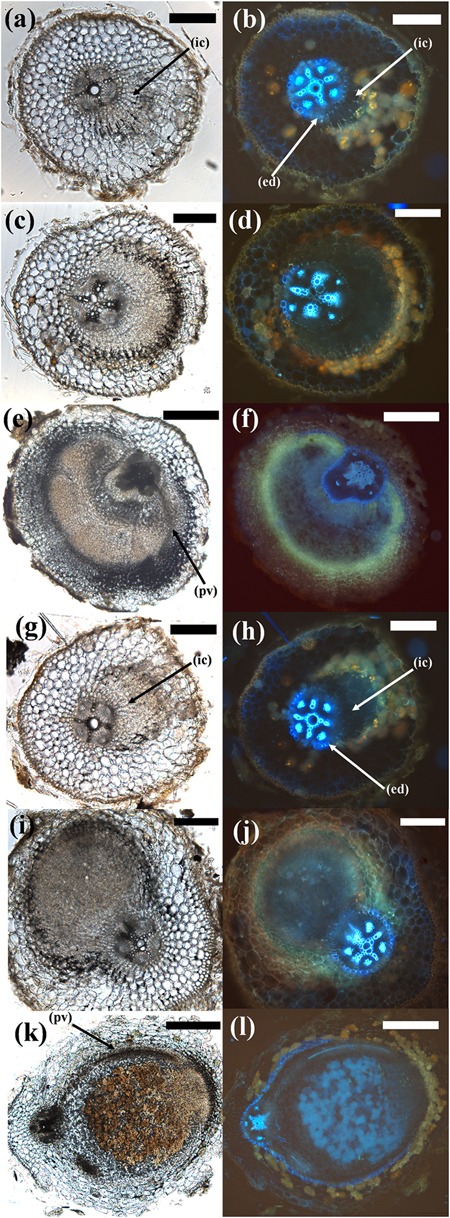
Comparison of different stages of pseudonodule and nodule development in *C. arietinum*. **(a–f)** *C. arietinum* roots treated with 10 μM BAP; **(g–l)** *C. arietinum* inoculated with *M. ciceri*. Sections on the left show the brightfield images, photos on the right side show the respective images under UV exposure to visualize flavonoids (yellow–orange or blue autofluorescent cell content mainly in cortical cells surrounding nodules/primordia) and the endodermis (strongly blue auto-fluorescent cell walls). (**a,b**; 5d post-BAP) and (**g,h**; 5d post-inoculation) Early-stage primordia showing first cell divisions in the inner cortex (ic) which can be distinguished from the vascular tissue and the pericycle by the fluorescent endodermis (ed). (**c,d**; 7d post-BAP) and (**i,j**; 7d post-inoculation) Medium-stage primordia. Note the more clearly defined structure of the inoculated phenotype. (**e,f**; 14d post-BAP) Mature pseudonodule displaying peripheral vasculature (pv). (**k,l**; 14d post-inoculation) Mature nodule displaying peripheral vasculature (pv). Bars **(a–d)**, **(g,h)** 200 μm; **(e,f)**, **(k,l)** 500 μm.

The developmental stages of nodules and pseudonodules were compared by examining cross-sections over a time course of 2–4 weeks in two species forming indeterminate nodules (*M. sativa, C. arietinum*) and two species forming determinate nodules (*L. japonicus, M. atropurpureum*), which were treated either with BAP or their respective symbionts. These species were chosen because they formed well-synchronized and well-differentiated pseudonodules at high frequencies. In *M. sativa* and *C. arietinum*, both BAP and rhizobia led to initiation of cell divisions mainly in the inner cortex that later developed into mature structures derived mainly from the root cortex (Figure 6 and Supplementary Figure 6). However, the final nodule shapes were somewhat different, with pseudonodules often either multi-lobed or broader than rhizobia-induced nodules (Figure 6 and Supplementary Figure 6). In both *M. sativa* and *M. truncatula*, roots containing pseudonodules often showed extensive differentiation of the vascular tissue (Supplementary Figure 6), although this was likely a result of natural root differentiation in older parts of the roots as it also occurred in untreated roots forming spontaneous nodules (Supplementary Figure 2).

**FIGURE 7:**
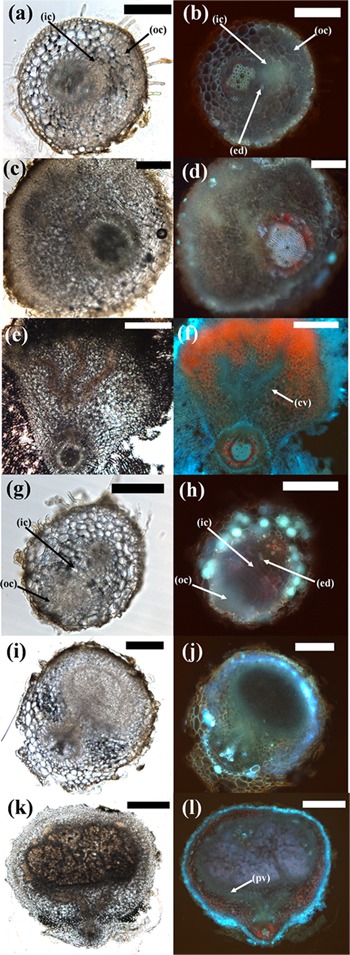
Comparison of different stages of pseudonodule and nodule development in *M. atropurpureum*. **(a–f)** *Macroptilium atropurpureum* roots treated with 10 μM BAP; **(g–l)** inoculated with *Sinorhizobium fredii* NGR234 strain ANU280. Sections on the left show the brightfield images, photos on the right side show the respective images under UV exposure to visualize the endodermis (light blue auto-fluorescent cell walls). (**a,b**; 7d post-BAP) and (**g,h**; 5d post-BAP) Early-stage primordia showing first cell divisions in the outer (oc) and inner cortex (ic) which can be distinguished from the vascular tissue and the pericycle by the fluorescent endodermis (ed). (**c,d**; 14d post-BAP) and (**i,j**; 10d post-inoculation) Medium-stage primordia showing divisions predominately originating from the outer cortex in both treatments. (**e,f**; 21d post-BAP) Mature pseudonodule displaying central vasculature (cv). (**k,l**; 14d post-inoculation) Mature nodule displaying peripheral vasculature (pv). Bars **(a–j)** 200 μm; **(k,l)** 500 μm. Red pigment is most likely due to chlorophyll autofluorescence, especially in the mature pseudonodule and vascular tissue.

In *L. japonicus* and *M. atropurpureum*, both BAP and rhizobia treatment lead to initiation of cell divisions in the outer as well as inner root cortex (Figure 7 and Supplementary Figure 7). Mature *M. atropurpureum* pseudonodules often contained multiple central vascular strands and nodule lobes compared to single lobed rhizobia-induced nodules with peripheral vasculature (Figures 7e,f,k,l). Chickpea pseudonodules were often broad-shaped and typically formed peripheral vasculature (Figure 6e). Infected nodules as well as pseudonodules often contained fluorescent flavonoids, which appear to be markers of cortical cell division across legumes (Mathesius et al., 1998; Figures 6, 7 and Supplementary Figures 6, 7). The effects of external nitrogen on the inhibition of pseudonodulation and nodulation were also assayed in four legume species (*C. arietinum, M. sativa, T. repens, M. atropurpureum*). *T. repens* was included in this experiment because a previous study had shown that nitrate addition only partially prevented *ENOD40* induction by BAP (Mathesius et al., 2000a), while nitrate inhibition of pseudonodules in *L. japonicus* had been demonstrated previously (Heckmann et al., 2011). All four species displayed significant reduction of nodule and pseudonodule numbers following addition of 10 mM KNO_3_ to the growth media (Supplementary Figure 8).

### Localizing Tissue Responses to Cytokinin in *A. thaliana TCSn::GFP* Reporter Plants

**FIGURE 8:**
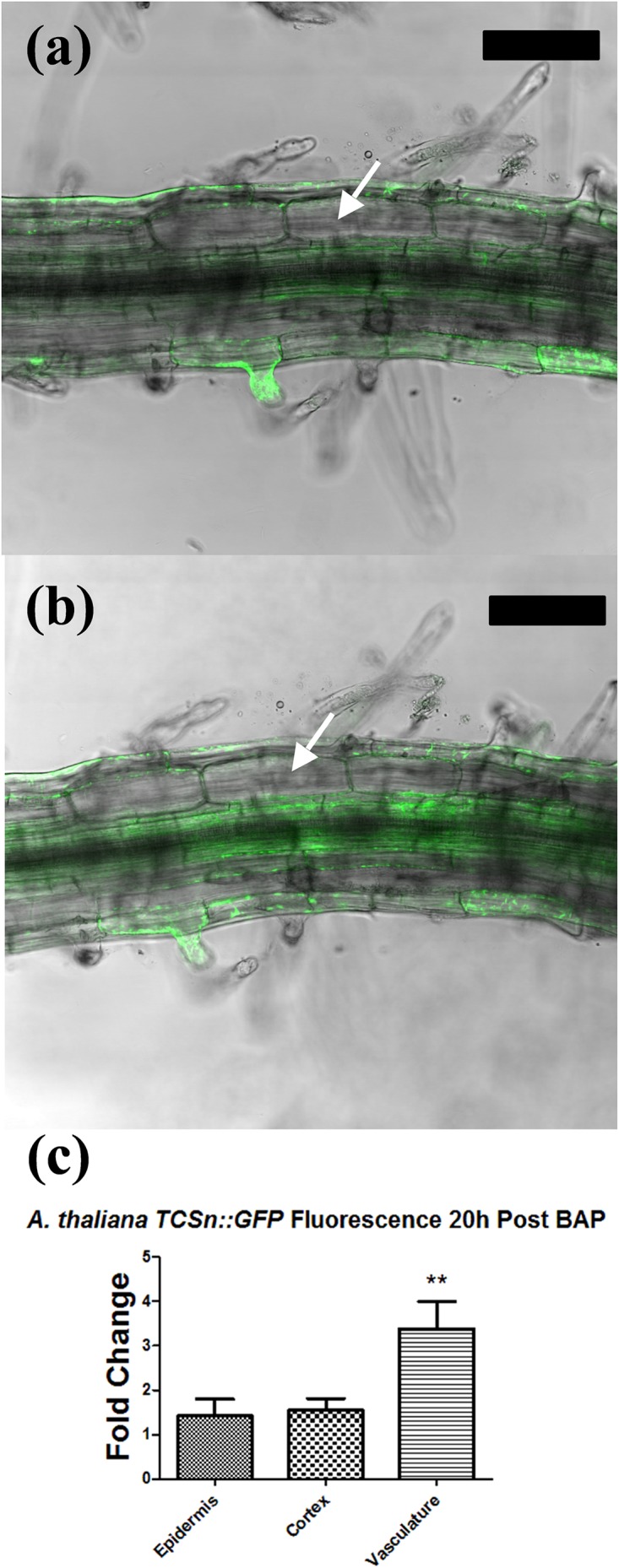
Tissue-specific response in *Arabidopsis thaliana* TCSn::GFP following cytokinin treatment. **(a,b)** Confocal laser scanning microscope images of *A. thaliana TCSn::GFP* root 1 cm behind the root tip. GFP fluorescence (green) is overlaid on the brightfield image. **(a)** Root before treatment. **(b)** Root after incubation in 1 μM BAP solution for 20 h. **(c)** Fold change increase in GFP fluorescence intensity in three different tissue types. Fold changes reflect the ratio of fluorescence intensity values before and after BAP treatment. Values were statistically compared using a two-tailed *t*-test. *n* = 6. ^∗∗^ indicates *p* < 0.01. White arrows, cortical cells. Bars, 100 μm.

The fluorescence of *A. thaliana* seedlings expressing the cytokinin reporter *TCSn::GFP* was recorded before and after 20 h incubation in 1 or 10 μM BAP. Visualization of whole seedling roots under a fluorescent stereomicroscope showed expression in the root apical meristem and the vascular tissue, which was enhanced in the same tissues after BAP application, as described in other studies (Zürcher et al., 2013; Supplementary Figure 9). A more detailed analysis was carried out using confocal microscopy. Six *A. thaliana* seedlings expressing the cytokinin reporter *TCSn::GFP* were analyzed at 1 mm from the root tip, the root–hair elongation zone where pseudonodules and nodules are typically initiated in legumes, before and after 20 h incubation in 1 μM BAP. Untreated roots showed GFP expression in vascular and epidermal cells, but not in cortical cells (Figure 8). The GFP expression was significantly increased in the vascular tissue following BAP application, but remained unchanged in the epidermis and was not induced in cortical cells (Figure 8).

## Discussion

### Cytokinin-Induced Pseudonodulation Is a Conserved Response Among Nodulating Legumes

Prior to this study, BAP-induced pseudonodulation had only been described in detail in three nodulating legume species; *M. sativa* (Hirsch et al., 1997), *T. repens* (Mathesius et al., 2000a), and *L. japonicus* (Heckmann et al., 2011). This study has shown that BAP-induced pseudonodulation is a conserved feature among nodulating legumes, and was observed in species from all three legume subfamilies. Pseudonodulation was also induced by the exogenous application of four endogenous cytokinins in *M. atropurpureum*, suggesting that this is not a BAP-specific response. A characteristic phylogenetic trait of Papilionoids is a 50-kb inversion in their genome, which occurred early in the diversification of Papilionoids (Cannon et al., 2014) and may have led to the diversification of nodulation-related genes, including cytokinin response regulators (Op den Camp et al., 2011). The functional consequences of this inversion are still not fully known. On account of the conserved response observed between species of each legume subfamily, it appears that cytokinin-induced phenotypes are not contingent upon the inheritance of the 50-kb inversion.

Cross-sectional analysis revealed that pseudonodules in all species display anatomical similarities with infected nodules, namely cortical cell divisions. In the two species forming determinate nodules, *L. japonicus* and *M. atropurpureum*, BAP treatment induced initial cell divisions in the outer and inner cortex, whereas in two species forming indeterminate nodules, *C. arietinum* and *M. sativa*, initial cell divisions were seen mainly in the inner cortex. These similarities in phenotypic response between pseudonodules and rhizobia-induced nodules suggest that the origin of nodule primordia is determined by a cell-specific cytokinin response.

In *M. pudica*, pseudonodules originated only from the junction of the main root with lateral roots and may reflect a “crack-entry” infection strategy common among Mimosoids (Sprent, 2009). Interestingly, peripheral vascularization and even pink pigmentation were prominent in pseudonodules of *C. arietinum*, which only appeared during late stages of development. The presence of vascular tissue in *C. arietinum* and *M. atropurpureum* pseudonodules contrasted with the noted absence of vascular tissue in *L. japonicus* pseudonodules reported here and by Heckmann et al. (2011). Another indication that pseudonodules share developmental similarities with infected nodules is that nitrogen in the growth media inhibited their development, consistent with previous observations (Heckmann et al., 2011).

In addition, the *M. truncatula cre1-1* mutant defective in the transduction of Nod factor-induced cytokinin signaling (Plet et al., 2011) did not form pseudonodules or cortical cell divisions. This indicates that the cytokinin-induced phenotype is dependent on the same cytokinin receptor involved in nodule development. Similarly, the *L. japonicus lhk1* cytokinin receptor mutant was shown not to form pseudonodules in response to BAP (Heckmann et al., 2011).

One role of cytokinin in plant development is as a negative regulator of root growth by inhibiting lateral root initiation in the pericycle (e.g., Laplaze et al., 2007) and inhibition of the root apical meristem (e.g., Rùžička et al., 2009). In view of the conserved pseudonodulation observed in nodulating legumes, it is important to consider whether this response is the result of a heightened general sensitivity to cytokinin, or rather a specific sensitivity. Cytokinin treatments resulted in a consistent reduction in root growth and lateral root formation across most species, irrespective of their propensity to form pseudonodules. This indicates that pseudonodulation is dependent on a positive effect of cytokinin in the root cortex, which is independent of inhibition of cell proliferation in the pericycle and root apical meristem.

Apart from differences in the observed cytokinin responses between nodulating and non-nodulating species, significant variation in cytokinin-induced pseudonodulation has been observed in different ecotypes of *L. japonicus* (Heckmann et al., 2011). A stark difference in response to cytokinin was also noted here between two closely related species: *M. sativa* and *M. truncatula*. Only *M. sativa* produced pseudonodules spontaneously, as previously reported by Joshi et al. (1991). Furthermore, pseudonodules produced by *M. sativa* displayed a more advanced degree of development compared to those produced in *M. truncatula*. This difference between closely related plants may be due to differential expression of cytokinin oxidases, which have been shown to play important roles in coordinating cytokinin responses during nodule development, and which could confer feedback inhibition to external cytokinin application (Reid et al., 2016). Additionally, different members of type-A response regulators in different species may differentially regulate cytokinin responses during nodulation (Gamas et al., 2017).

### Pseudonodules Were Not Observed in Plant Species Unable to Form Cortical-Based Nodules

**FIGURE 9:**
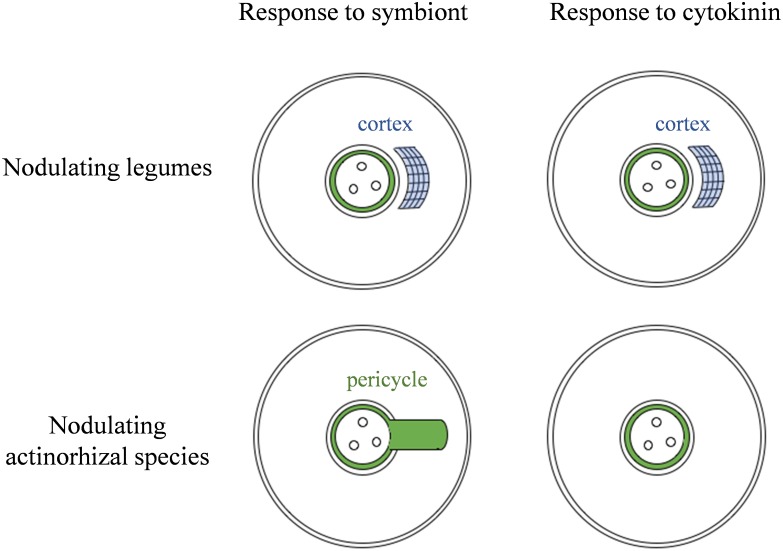
Model for the observed correlation between the site of nodule organogenesis and cortical response to cytokinin. Nodulating legumes typically form nodules originating predominantly in the cortex, and this correlates with the ability to proliferate cortical cells in response to cytokinin, which then leads to pseudonodule formation. Actinorhizal species that nodulate with *Frankia* spp. typically form nodules from modified lateral roots. These species lack cortical cytokinin responses and pseudonodule formation.

RNS is not a unique feature of legumes, as it is found in numerous species belonging to the other three orders that make up the Fabid clade. These non-legume RNS plants recruit actinorhizal bacteria as their nitrogen-fixing symbiont, as opposed to legumes which recruit rhizobia. In this study, we have shown that nodulating non-legume Fabids (*A. glutinosa, C. glauca*, and *D. glomerata*) produced neither pseudonodules nor cortical cell divisions following exogenous cytokinin treatment. This differential response may indicate key anatomical differences between rhizobial and actinorhizal nodules (Figure 9). Actinorhizal nodules originate from the pericycle and are morphologically akin to lateral roots (Pawlowski and Sprent, 2007) and as such may implement a different signaling pathway to initiate nodule organogenesis, even though some of the early symbiotic signaling pathway is shared with legumes (Svistoonoff et al., 2014).

We note that our results contradict previously published findings which reported the development of pseudonodules in *A. glutinosa* (Rodriguez-Barrueco and Bermudez De Castro, 1973) and *N. tabacum* (Arora et al., 1959) following treatment with kinetin. In our study, exogenous treatment with BAP did not result in organogenesis despite root growth being significantly inhibited in both species. Treatment with four endogenous cytokinins including kinetin also did not result in organogenesis, although cell-swelling resulted in *A. glutinosa* following treatment with *cis*-zeatin. This discrepancy may be due to differences in experimental design. Rodriguez-Barrueco and Bermudez De Castro (1973) grew plants in hydroponics with roots exposed to a constant concentration of cytokinin for several weeks. Arora et al. (1959) propagated plants via tissue culture and also grew them in hydroponics with roots exposed to a constant source of kinetin. Propagation via tissue culture can induce genetic changes in plants (e.g., Phillips et al., 1994) and it is possible that the growth conditions induced a phenotypic response not generally observed under natural growth conditions. Arora and colleagues also noted that the structures displayed considerable morphological differences compared to legume nodules in that they were largely undifferentiated and always associated with the induction of lateral roots. We cannot rule out the possibility that lack of pseudonodule formation observed in many species in our study was due to our specific growth conditions, nevertheless, we conclude that the non-nodulating species we examined are either unable to form pseudonodules, or differ significantly from nodulating legumes in their sensitivity to the action of cytokinin in the cortex.

A surprising result was the observation that *H. annuus* (sunflower) responded to cytokinin with limited cortical cell divisions, although these did not proceed to give rise to primordia, as was observed in all nodulating legume species. It is possible that the cytokinin response in *H. annuus* is rapidly downregulated after cytokinin addition or that cytokinin addition activates its degradation, similar to observations in *L. japonicus* (Reid et al., 2016). Alternatively, the cytokinin response observed in *H. annuus* may be lacking elements necessary for transition of cortical cell divisions to primordium formation (Tirichine et al., 2007; Heckmann et al., 2011). Other non-legume species are capable of activating cortical cell divisions, for example during lateral root development, but these do not proceed to form nodule primordia (McCully, 1975). This includes members of the Rosids related to actinorhizal species, e.g., *C. pepo* (Ilina et al., 2018) as well as legumes (Mathesius et al., 2000b; Herrbach et al., 2014).

Evidence from phylogenetic studies suggests that non-nodulating legume species have lost the ability to form RNS (Sprent, 2009), in one of many possible ways. With environmental changes leading to increased soil nitrogen, the carbon-cost associated with RNS may have outweighed the benefits. Many plant pathogens exploit the cytokinin pathway of plants either through direct synthesis of cytokinin or by altering the plant host’s cytokinin pathway (Giron et al., 2013) and therefore the costs associated with pathogenicity may have played a part in driving the loss of a specific cytokinin sensitivity. Comparative phylogenomics studies have so far not identified any genes specific to nodulating plants (Griesmann et al., 2018) and of the few that could explain loss of nodulation in non-nodulators, the main candidates are *NIN* and *Rhizobium-directed Polar Growth* (*RPG*). *RPG* is required for infection and thus unlikely to be associated with pseudonodule formation. NIN is essential for infection and cortical cell division in legumes, and for pseudonodule formation in *L. japonicus* (Heckmann et al., 2011), and it interacts with cytokinin signaling in the cortex through a positive feedback loop (Gamas et al., 2017). *NIN* has been lost in several non-nodulating species, including *C. australe* (Griesmann et al., 2018), which was included in this study. However, *NIN* is unlikely to predict the ability for pseudonodule formation because it was shown to be necessary for the formation of nodules in actinorhizal species, which did not form pseudonodules in our study, and do not show cortical-based nodule development (Svistoonoff et al., 2014). To our knowledge, no other candidate genes are currently known that could explain the differential ability of nodulating legumes to form pseudonodules.

### *A. thaliana* Lacks a Cortical Cytokinin Response Following BAP Treatment

Since *A. thaliana* was shown in prior assays to not respond to cytokinin with induction of cortical-cell divisions it was hypothesized that the cortex cannot perceive cytokinin. To investigate this possibility, the spatial profile of cytokinin perception was analyzed in *A. thaliana TCSn::GFP* reporter plants, which revealed that cytokinin elicited a response in the vascular tissue, but not in the cortical cell layer. In contrast, the *TCSn::GUS* reporter was induced in the cortex of *M. truncatula* (Fonouni-Farde et al., 2017) and *L. japonicus* (Reid et al., 2017) in response to cytokinin. One possible explanation for why some plants are able to respond to cytokinin with the initiation of pseudonodules, while others cannot, is that cytokinin is either not perceived or not translated into the required downstream responses by the cortex in non-responders. Differences in expression or localization of cytokinin response genes may account for the lack of cortical response in non-nodulating plants. An expansion of the cytokinin response pathway is a noted feature of land plant evolution (Pils and Heyl, 2009), and this might extend to a novel cytokinin perception module in the cortex of nodulating legumes, correlating with their unique ability to initiate nodules *de novo* from cortical cells. Future studies will have to identify the nature of such a proposed cortex-specific cytokinin response in nodulating legumes, and its suggested loss in non-nodulating legumes.

## Author Contributions

All authors contributed to the conception of the study and writing of the manuscript. CG-C carried out experimental work with contributions from RW and UM.

## Conflict of Interest Statement

The authors declare that the research was conducted in the absence of any commercial or financial relationships that could be construed as a potential conflict of interest.

## Footnotes

1 https://imagej.nih.gov/ij/
