# Supplementary material for: Nodulating Legumes Are Distinguished by a Sensitivity to Cytokinin in the Root Cortex Leading to Pseudonodule Development
Source: Front Plant Sci. 2019 Jan 8;9:1901. doi: 10.3389/fpls.2018.01901 (PMC6331541; doi:10.3389/fpls.2018.01901)
Supplement: Supplementary file 1 [file Data_Sheet_1.docx]

Supplementary Material

Nodulating legumes are distinguished by a sensitivity to cytokinin in the root cortex leading to pseudonodule development

Christopher Gauthier-Coles, Rosemary White, Ulrike Mathesius*

*** Correspondence:** ulrike.mathesius@anu.edu.au

**
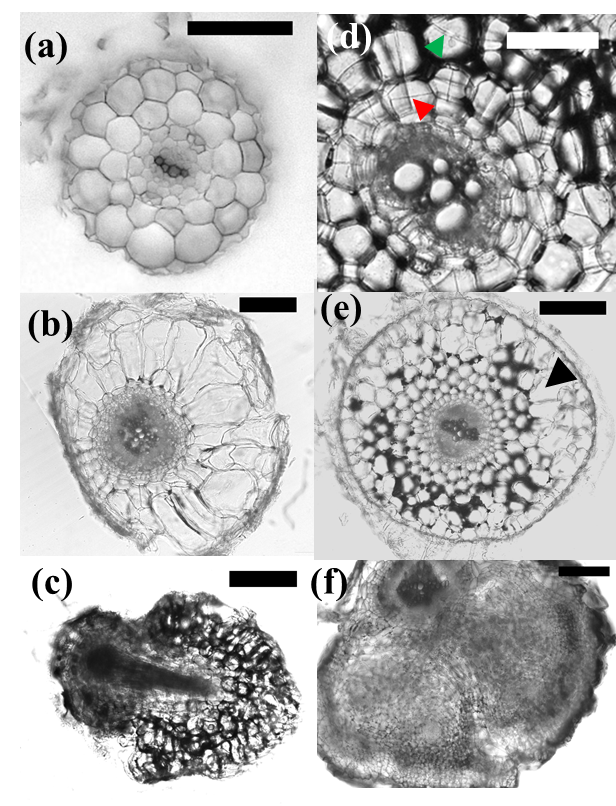
**

**Supplementary Figure 1.** *Range of different BAP induced anatomical phenotypes*

(a) *Nicotiana tabacum* treated with 10μM BAP displaying no apparent phenotypic response. **b:**  *Medicago truncatula* treated with 1μM BAP displaying cortical cell swelling. **c:** *Alnus glutinosa* treated with 50μM cis-zeatin displaying cortical cell swelling in an emerging lateral root. **d:** *Helianthus annuus* treated with 20μM BAP displaying limited cell divisions in inner (red arrow) and outer cortex (green arrow). **e:** *cre1-1* mutant treated with 1μM BAP showing no sign of cell division, however cell-swelling is still present (black arrow). **f:** Mature pseudonodule in *Macroptilium atropurpureum* treated with 10μM BAP. Bars (a-e) 200 μm, (e) 500 μm.


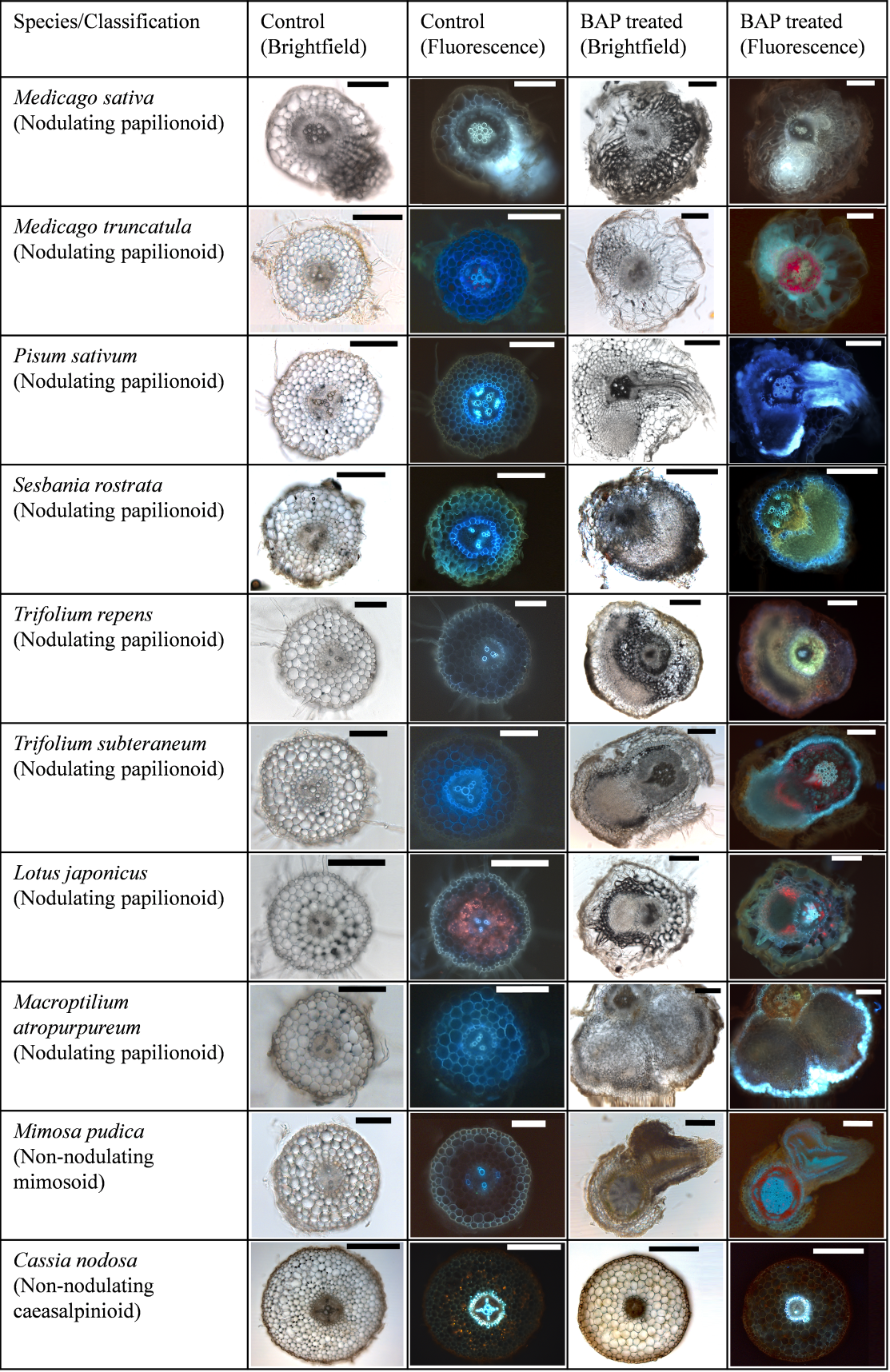


**Supplementary Figure 2.** *Cytokinin leads to pseudonodule development only in nodulating legume species*

The left columns of control and treated roots shows bright field images, while the right side shows fluorescent images after illumination under UV to visualise the autofluorescent endodermis. *M. sativa* 10μM BAP (Bars C [control] 200μm; T [treated] 200μm); *M. truncatula* 1μM BAP (Bars C 200μm; T 200μm); *P. sativum* 10μM BAP (Bars C 200μm; T 500μm); *S. rostrata* 10μM BAP (Bars C 200μm; T 200μm); *T. repens* 10μM BAP (Bars C 100μm; T 200μm); *T. subteraneum* 10μM BAP (Bars C 100μm; T 200μm); *L. japonicus* 10μM BAP (Bars C 200μm; T 200μm); *M. atropurpureum* 10μM BAP (Bars C 200μm; T 200μm); *M. pudica* 1μM BAP (Bars C 100μm; T 200μm); *C. nodosa* 10μM BAP (Bars C 500μm; T 200μm).


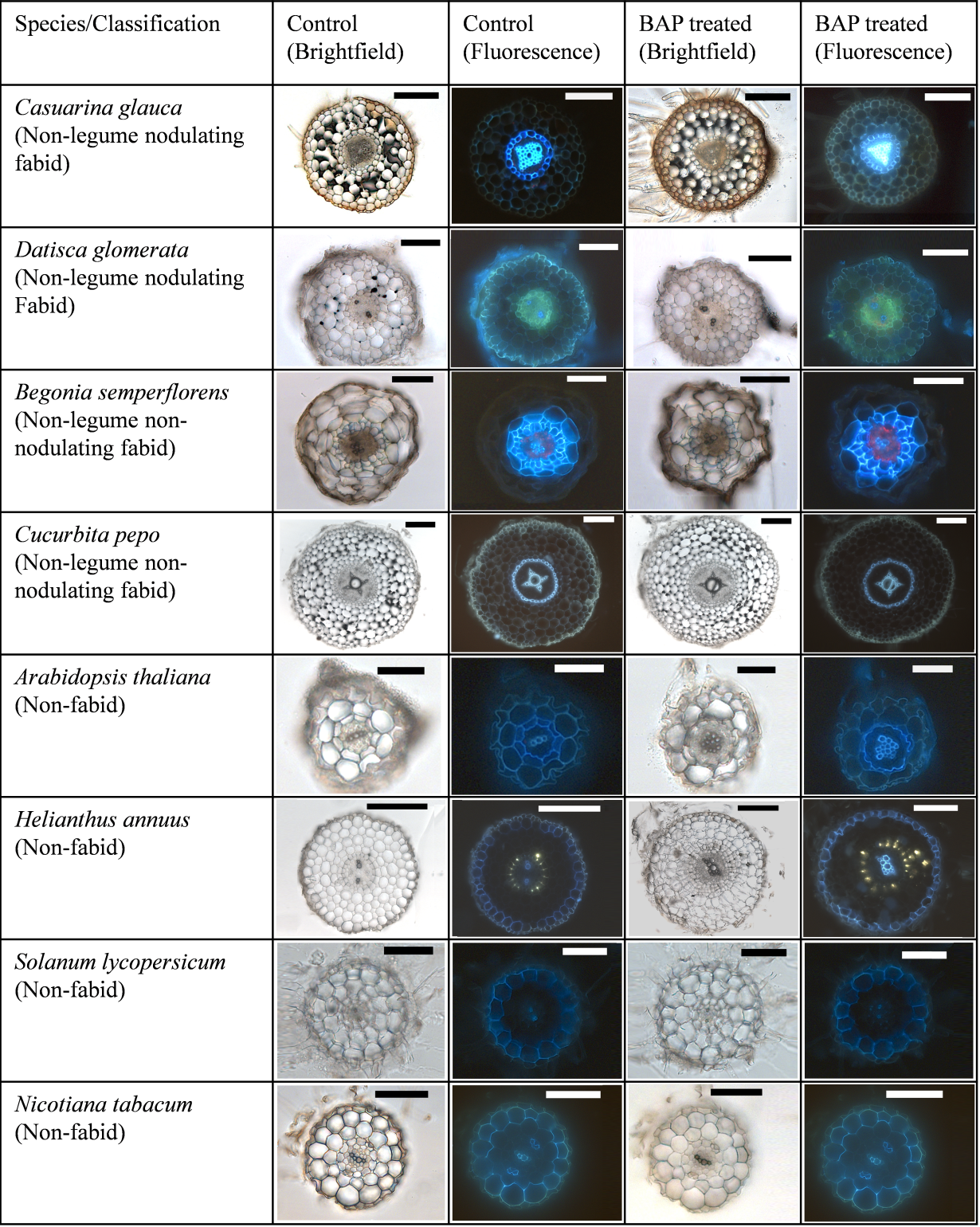


**Supplementary Figure 3.** *Cytokinin-induced organogenesis is not observed in non-legumes*

*C. glauca* 10μM BAP (Bars 100μm); *D. glomerata* 10μM BAP (Bars 100μm); *B. semperflorens* 10μM BAP (Bars 100μm); *C. pepo* 10μM BAP (Bars 200μm); *A. thaliana* 10μM BAP (Bars 50μm);  *H. annuus* 20μM BAP (Bars 200μm); *S. lycopersicum* 40μM BAP (Bars 100μm); *N. tabacum* 10μM BAP (Bars 100μm). The left columns of control and treated roots shows bright field images, while the right side shows fluorescent images after illumination under UV to visualize the autofluorescent endodermis (although this is not distinct in *L. lycopersicum* and *N. tabacum*).


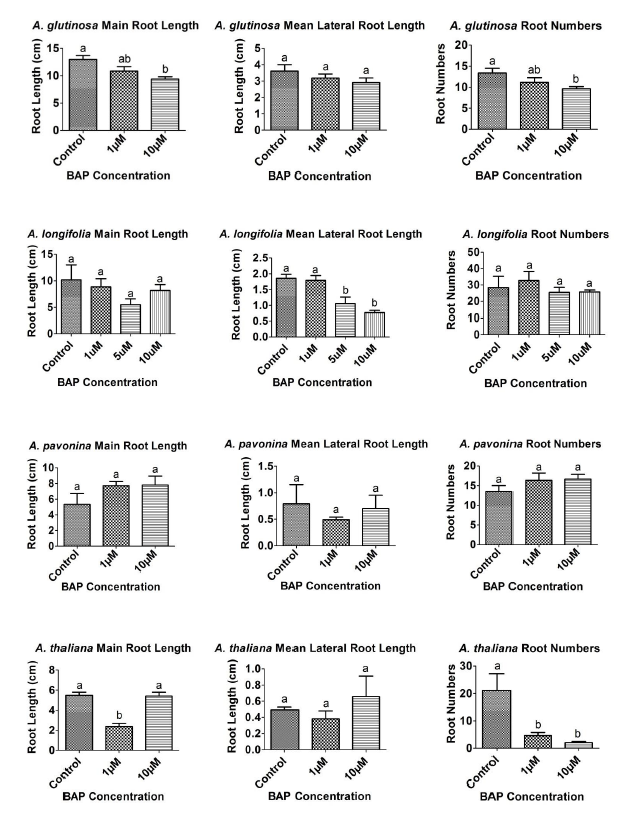


**Supplementary Figure 4.** *Root lengths and numbers of BAP-treated plants grown on plates*

For each of the three parameters tested, one-way analysis of variance with Tukey post-test was performed to statistically compare treatments. Different lowercase letters indicate significant differences at p<0.05. Graphs show means and standard error. Refer to Table S2 for replicate numbers.


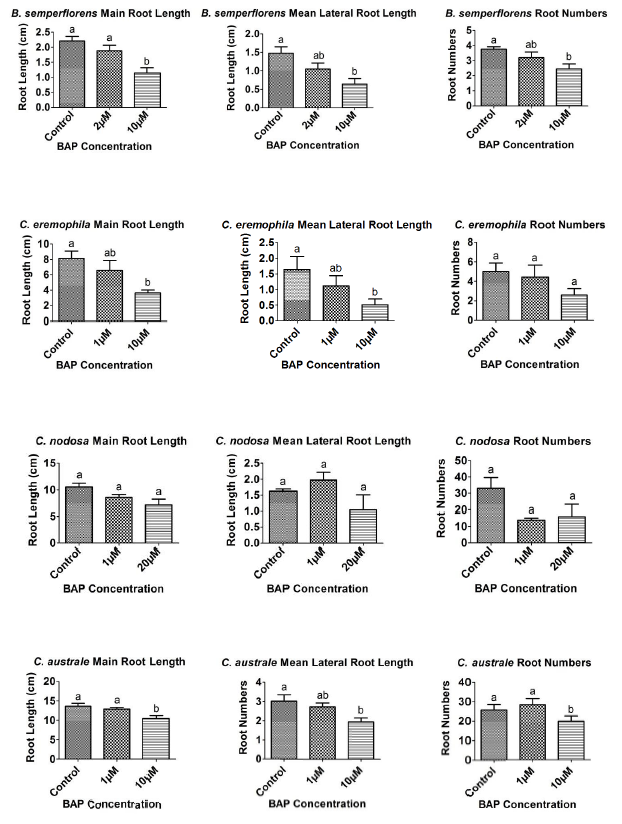


**Supplementary Figure 4.** ***Cont.***


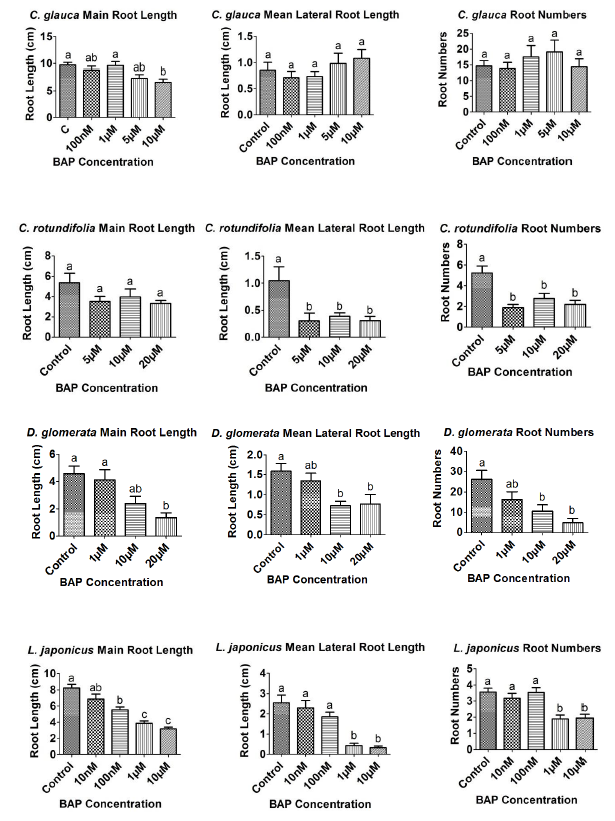


**Supplementary Figure 4.** ***Cont.***


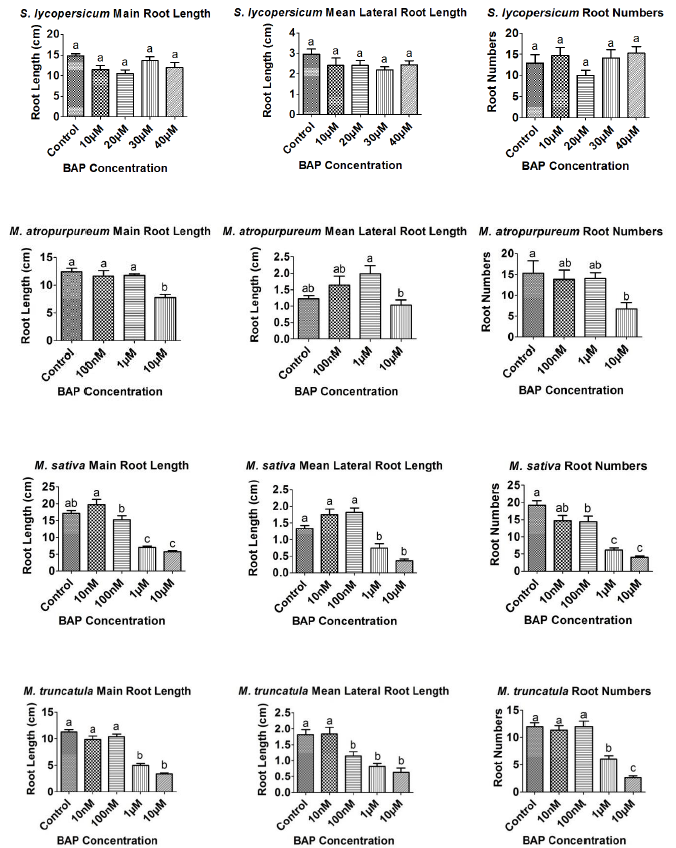


**Supplementary Figure 4.** ***Cont.***


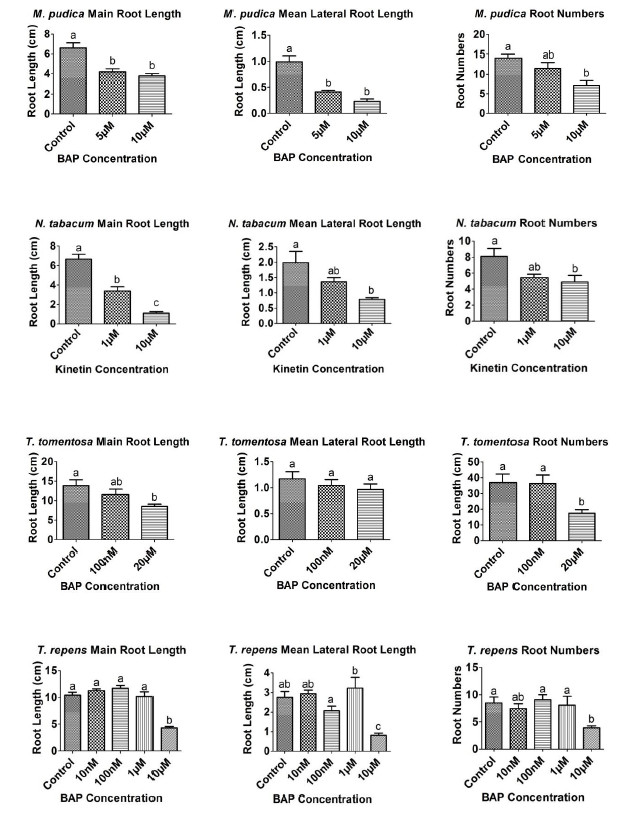


**Supplementary Figure 4.** ***Cont.***


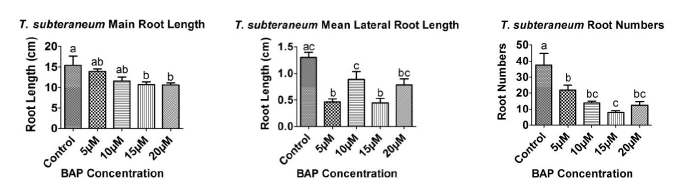


**Supplementary Figure 4.** *Cont.*


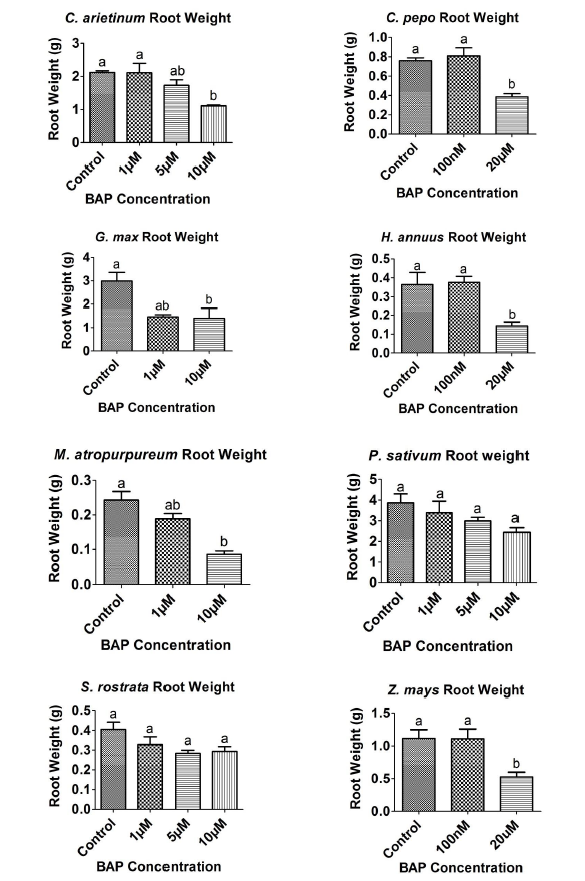


**Supplementary Figure 5.** *Root weights* *of BAP-treated plant species grown in pots*.

Because of the complex root architecture of pot-grown plants, root weight was used as a proxy for changes in root length due to BAP treatment. One-way analysis of variance with Tukey post-test was performed to statistically compare treatments. Different lowercase letters indicate significant differences at p<0.05. Graphs show means and standard error. Refer to Table S2 for replicate numbers.


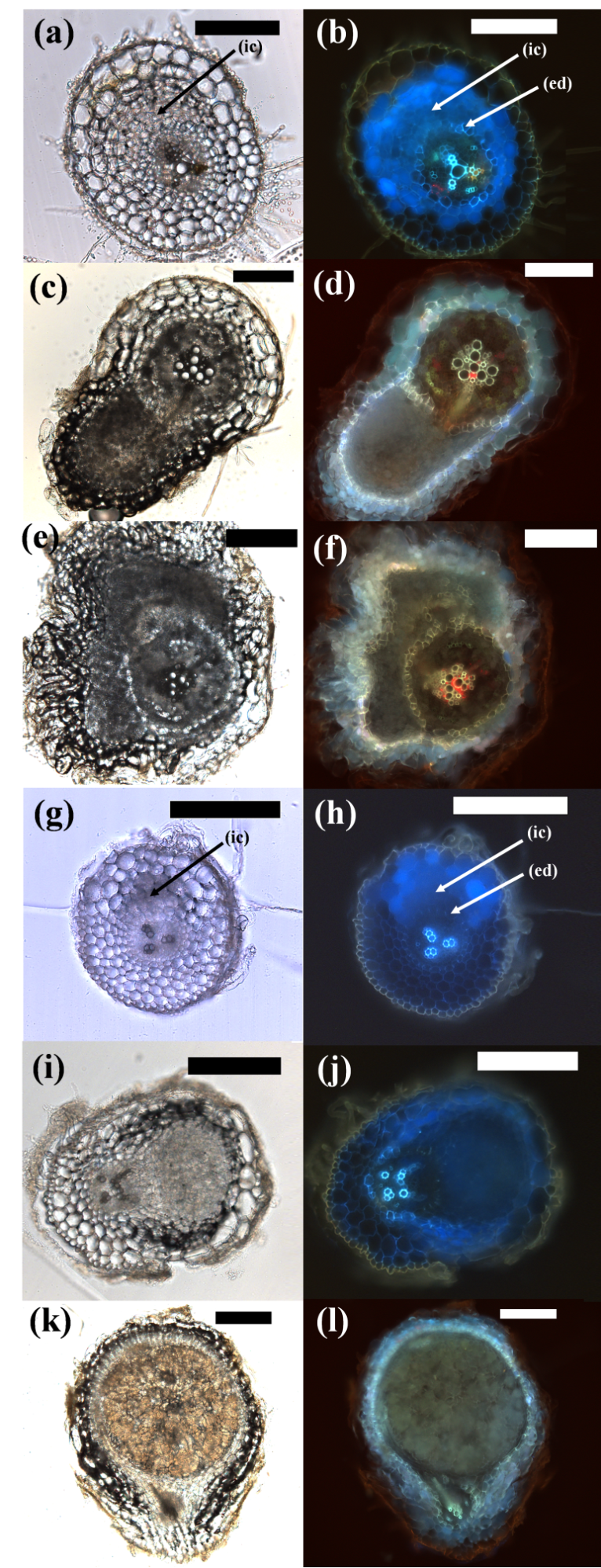


**Supplementary Figure 6.** *Comparison of different stages of pseudonodule and nodule development in* Medicago sativa:

(a-f) *M. sativa* roots treated with 1μM BAP; (g-l) inoculated with *Sinorhizobium meliloti*. Sections on the left show the bright field images; photos on the right side show the respective images under UV exposure to visualize flavonoids and the endodermis. (a, b; 4d post-BAP) and **(**g, h; 4d post-inoculation). Early-stage primordia showing first cell divisions in the inner cortex (ic) which can be distinguished from the vascular tissue and the pericycle by the fluorescent endodermis (ed). (c, d; 7 post-BAP) and (i, j; 7d post-inoculation**)** Medium-stage primordia. (e, f; 14d post-BAP) Mature pseudonodule. (k, l; 14d post-inoculation) Mature nodule. Bars (a-j) 200 μm; (k,l) 500 μm.


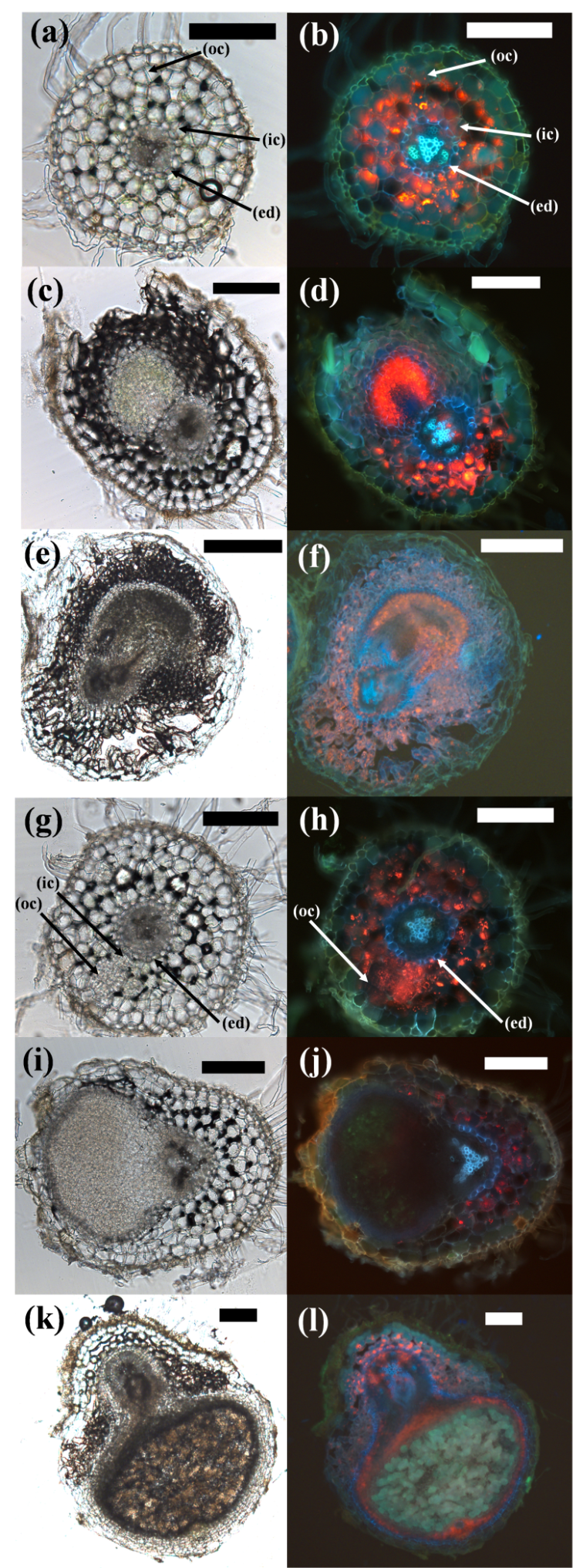


**Supplementary Figure 7.** *Comparison of different stages of pseudonodule and nodule development in* Lotus japonicus:

(a-f) *L. japonicus* roots treated with 1μM BAP; (g-l) inoculated with *Mesorhizobium loti*. Sections on the left show the bright field images; photos on the right side show the respective images under UV exposure to visualize the endodermis. (a, b; 5d post-BAP) and (g, h; 7d post-inoculation) Early-stage primordia showing first cell divisions in the inner cortex (ic), which can be distinguished from the vascular tissue and the pericycle by the fluorescent endodermis (ed). (c, d; 14d post-BAP**)** and **(**i, j; 14d post-inoculation) Medium-stage primordia. Note the more clearly defined structure of the inoculated root. (e, f; 21d post-BAP) Mature pseudonodule. (k, l; 21d post-inoculation) Mature nodule. Bars (a-j) 200 μm; (k, l) 500 μm. Red fluorescence is most likely due to the presence of chlorophyll in the plate-grown roots and was commonly observed in *L. japonicus*.


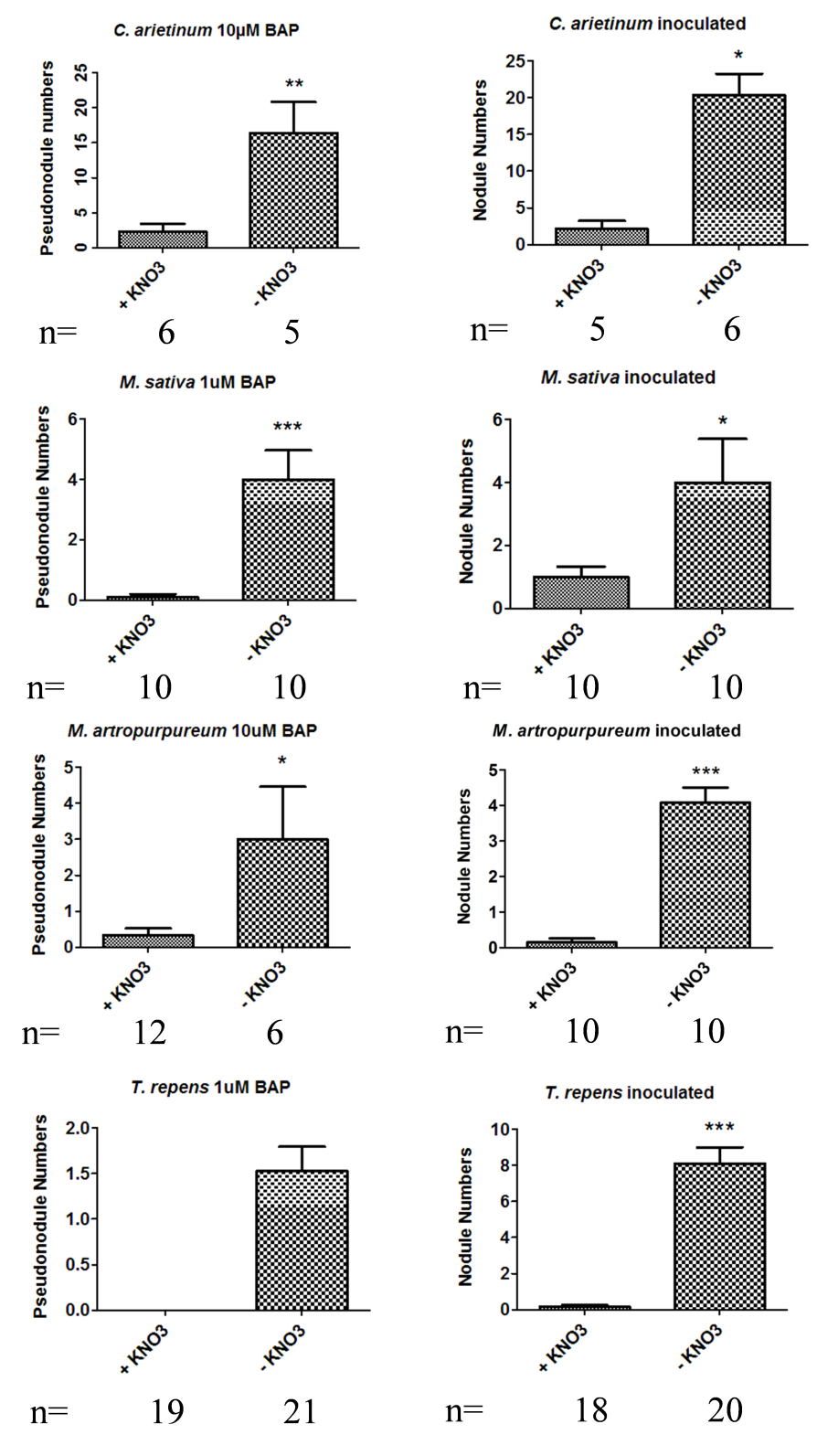


**Supplementary Figure 8.** *Effects of added nitrogen on the numbers of nodules and pseudonodules*

A Student’s *t* test was used for statistical analyses between plants grown in nitrogen containing media (10 mM KNO_3_) and plants grown in nitrogen-free media. Asterisks indicate significant differences in pseudonodule and nodule numbers per plant. (* P<0.05, **P < 0.01 and ***P < 0.001). Graphs show means and standard deviations, numbers of replicates (n) shown in figure.


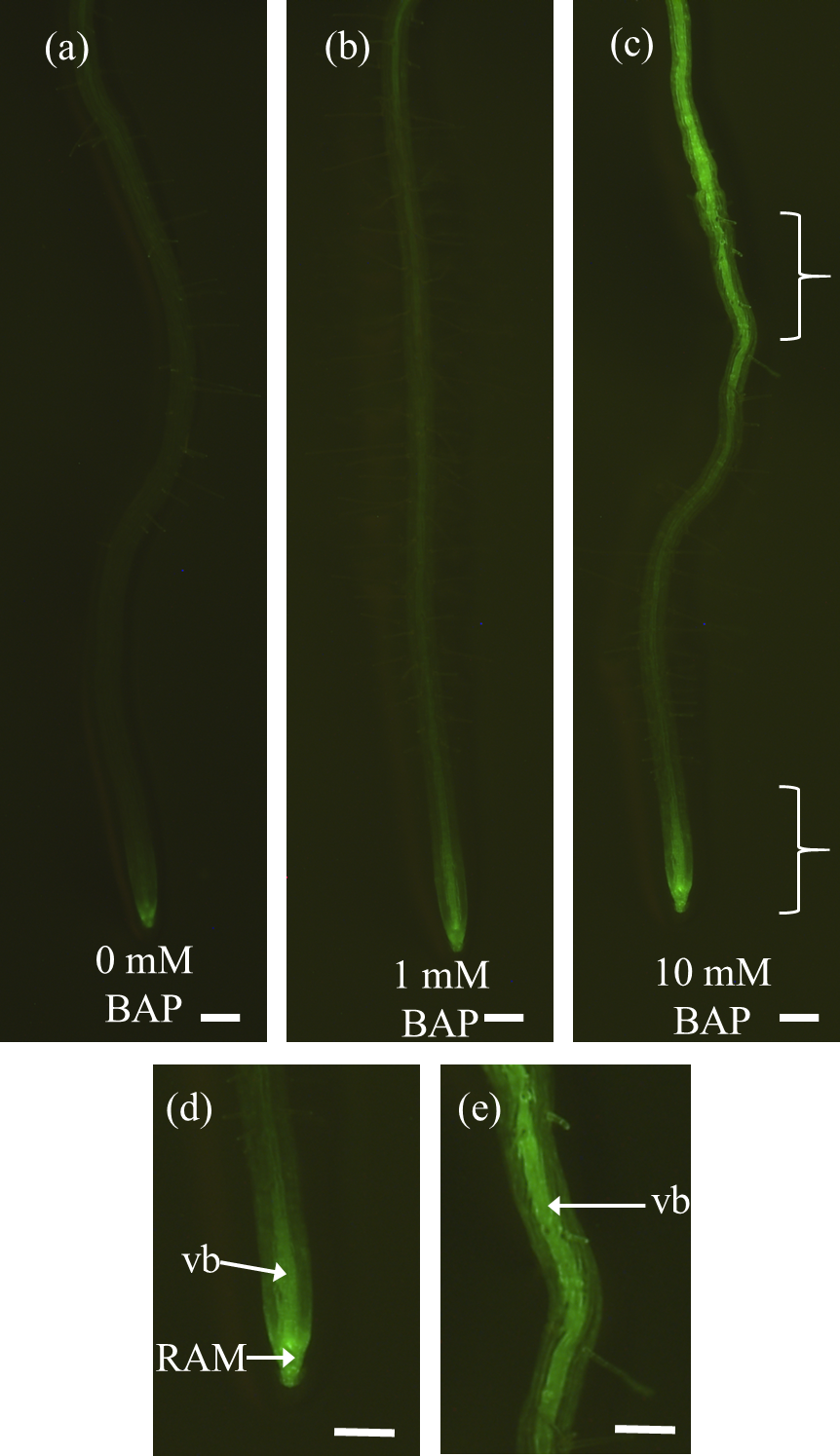


**Supplementary Figure 9.** TCSn:GFP *expression in* Arabidopsis thaliana *in response to BAP*.

Five day-old seedlings were treated with 0 (a), 1 (b) or 10 μM (c) BAP for 24 h and visualized under a fluorescence stereomicroscope to localize cytokinin responses. Root sectors indicated in (c) are shown at higher magnification in (d) and (e). Responses were seen around the root apical meristem (RAM) and vascular bundle (vb). Images are examples of five to ten replicates each taken with identical exposure settings.

|  | Species | Common name | Cultivar | Pre-Treatment | Growth Media |
| --- | --- | --- | --- | --- | --- |
| Nodulating legumes | *Acacia longifolia* | N/A | N/A | Heat | Fåhraeus Agar |
|  | *Chamaecrista rotundifolia* | Wynn Cassia | N/A | N/A | Fåhraeus Agar |
|  | *Cicer arietinum* | Chickpea | PBA Slasher | Soaking | Vermiculite |
|  | *Glycine max* | Soy Bean | N/A | Soaking | Vermiculite |
|  | *Lotus japonicus* | N/A | Gifu | Stratification | Fåhraeus Agar |
|  | *Macroptilium atropurpureum* | Siratro | N/A | N/A | Fåhraeus Agar |
|  | *Medicago sativa* | Lucerne | Aurora | N/A | Fåhraeus Agar |
|  | *Medicago truncatula* | Barrel Medic | Jemalong A-17 | Cold | Fåhraeus Agar |
|  | *Medicago truncatula cre1-1* | Barrel Medic | Jemalong A-17 | Acid | Fåhraeus Agar |
|  | *Mimosa pudica* | N/A | N/A | Heat | Fåhraeus Agar |
|  | *Pisum sativum* | Green Pea | Percy | Soaking | Vermiculite |
|  | *Sesbania rostrata* | N/A | N/A | N/A | Vermiculite |
|  | *Trifolium repens* | White Clover | N/A | N/A | Fåhraeus Agar |
|  | *Trifolium subteraneum* | Sub Clover | Karridale | N/A | Fåhraeus Agar |
| Non-nodulating legumes | *Adenanthera pavonina* | N/A | N/A | Acid | Vermiculite |
|  | *Cassia eremophila* | N/A | N/A | Heat | Vermiculite |
|  | *Cassia nodosa* | N/A | N/A | Acid | Vermiculite |
|  | *Castanospermum australe* | N/A | N/A | Soaking | Vermiculite |
| Nodulating non-legume fabids | *Alnus glutinosa* | Common Alder | N/A | N/A | Fåhraeus Agar |
|  | *Casuarina glauca* | N/A | N/A | N/A | Fåhraeus Agar |
|  | *Cucurbita pepo* | Zucchini | Black Jack | N/A | Fåhraeus Agar |
|  | *Datisca glomerata* | False Hemp | N/A | N/A | Hoagland’s Agar |
| Non-nodulating non-legume fabids | *Begonia semperflorens* | N/A | Cocktail Brandy | N/A | Fåhraeus Agar (0.5mM KNO_3_) |
|  | *Trema tomentosa* | N/A | N/A | N/A | Vermiculite |
| Non-fabids | *Arabidopsis thaliana* | Arabidopsis | Columbia-0 | Stratification | Fåhraeus Agar |
|  |  |  |  |  | (0.5mM KNO_3_) |
|  | *Arabidopsis thaliana TCSn::GFP* | Arabidopsis | Columbia-0 | Stratification | Fåhraeus Agar |
|  | *Helianthus annuus* | Sunflower | Giant Single | N/A | Vermiculite |
|  | *Nicotiana tabacum* | Tobacco | Wisconsin-38 | N/A | Fåhraeus Agar  (0.5mM KNO_3_) |
|  | *Solanum lycopersicum* | Tomato | Grosse Lisse | N/A | Fåhraeus Agar |
|  | *Zea mays* | Corn | Kelvedon Glory | N/A | Vermiculite |

**Supplementary Table1.** *List of subject species and respective germination treatments and growth media.*

| *Acacia longifolia* | Ratio of replicates producing pseudonodules | | | | | | | | | | | |
| --- | --- | --- | --- | --- | --- | --- | --- | --- | --- | --- | --- | --- |
|  | Control | 2µM | | | | | 5µM | | 10µM | | | 20µM |
|  | 0/10 | 5/10* | | | | | 5/10* | | 3/10 | | | 5/10* |
|  | Average number of pseudonodules per replicate | | | | | | | | | | | |
|  | 2µM | | 5µM | | | | | 10µM | | | | 20µM |
|  | 0.6 | | 1.2 | | | | | 0.8 | | | | 0.8 |
| *Adenanthera pavonina* | Ratio of replicates producing pseudonodules | | | | | | | | | | | |
|  | Control | | | | 1µM | | | | | 10µM | | |
|  | 0/10 | | | | 0/10 | | | | | 0/10 | | |
|  | Average number of pseudonodules per replicate | | | | | | | | | | | |
|  | NA | | | | | | | | | | | |
| *Alnus glutinosa* | Ratio of replicates producing pseudonodules | | | | | | | | | | | |
|  | Control | | | | 1µM | | | | | 10µM | | |
|  | 0/10 | | | | 0/10 | | | | | 0/10 | | |
|  | Average number of pseudonodules per replicate | | | | | | | | | | | |
|  | NA | | | | | | | | | | | |
| *Arabidopsis thaliana* | Ratio of replicates producing pseudonodules | | | | | | | | | | | |
|  | Control | | | 1µM | | | | | | 10µM | | |
|  | 0/10 | | | 0/10 | | | | | | 0/10 | | |
|  | Average number of pseudonodules per replicate | | | | | | | | | | | |
|  | NA | | | | | | | | | | | |
| *Begonia semperflorens* | Ratio of replicates producing pseudonodules | | | | | | | | | | | |
|  | Control | | | | 2µM | | | | | 10µM | | |
|  | 0/10 | | | | 0/10 | | | | | 0/10 | | |
|  | Average number of pseudonodules per replicate | | | | | | | | | | | |
|  | NA | | | | | | | | | | | |
| *Cassia eremophila* | Ratio of replicates producing pseudonodules | | | | | | | | | | | |
|  | Control | | | | 1µM | | | | | 10µM | | |
|  | 0/10 | | | | 0/10 | | | | | 0/10 | | |
|  | Average number of pseudonodules per replicate | | | | | | | | | | | |
|  | NA | | | | | | | | | | | |
| *Cassia nodosa* | Ratio of replicates producing pseudonodules | | | | | | | | | | | |
|  | Control | | | | | 1µM | | | | | 10µM | |
|  | 0/5 | | | | | 0/10 | | | | | 0/10 | |
|  | Average number of pseudonodules per replicate | | | | | | | | | | | |
|  | NA | | | | | | | | | | | |
| *Castanospermum australe* | Ratio of replicates producing pseudonodules | | | | | | | | | | | |
|  | Control | | | | | 1µM | | | | | 10µM | |
|  | 0/10 | | | | | 0/10 | | | | | 0/10 | |
|  | Average number of pseudonodules per replicate | | | | | | | | | | | |
|  | NA | | | | | | | | | | | |

**Supplementary Table 2.** *Pseudonodulation rate for each species tested species following exogenous BAP treatment.* *: chi-square significance (p<0.05) between treatments and control.

| *Casuarina glauca* | Ratio of replicates producing pseudonodules | | | | | | | | | | | | | | | |
| --- | --- | --- | --- | --- | --- | --- | --- | --- | --- | --- | --- | --- | --- | --- | --- | --- |
|  | Control | | | | | 1µM | | | | | | | 10µM | | | |
|  | 0/10 | | | | | 0/10 | | | | | | | 0/10 | | | |
|  | Average number of pseudonodules per replicate | | | | | | | | | | | | | | | |
|  | NA | | | | | | | | | | | | | | | |
| *Chamaecrista rotundifolia* | Ratio of replicates producing pseudonodules | | | | | | | | | | | | | | | |
|  | Control | 5µM | | | | | | 10µM | | | | 15µM | | | | 20µM |
|  | 0/10 | 5/10* | | | | | | 5/10* | | | | 4/10* | | | | 5/10* |
|  | Average number of pseudonodules per replicate | | | | | | | | | | | | | | | |
|  | 5µM | | 10µM | | | | | | | 15µM | | | | | | 20µM |
|  | 0.7 | | 1.5 | | | | | | | 0.5 | | | | | | 1.5 |
| *Cicer arietinum* | Ratio of replicates producing pseudonodules | | | | | | | | | | | | | | | |
|  | Control | | | 1µM | | | | | | | 5µM | | | | | 10µM |
|  | 0/3 | | | 3/3* | | | | | | | 3/3* | | | | | 3/3* |
|  | Average number of pseudonodules per replicate | | | | | | | | | | | | | | | |
|  | 1µM | | | | | | 5µM | | | | | | | | 10µM | |
|  | 7.7 | | | | | | 14.4 | | | | | | | | 27.3 | |
| *Cucurbita pepo* | Ratio of replicates producing pseudonodules | | | | | | | | | | | | | | | |
|  | Control | | | | | 1µM | | | | | | | 10µM | | | |
|  | 0/5 | | | | | 0/5 | | | | | | | 0/5 | | | |
|  | Average number of pseudonodules per replicate | | | | | | | | | | | | | | | |
|  | NA | | | | | | | | | | | | | | | |
| *Datisca glomerata* | Ratio of replicates producing pseudonodules | | | | | | | | | | | | | | | |
|  | Control | | | | | 1µM | | | | | | | 10µM | | | |
|  | 0/10 | | | | | 0/10 | | | | | | | 0/10 | | | |
|  | Average number of pseudonodules per replicate | | | | | | | | | | | | | | | |
|  | NA | | | | | | | | | | | | | | | |
| *Glycine max* | Ratio of replicates producing pseudonodules | | | | | | | | | | | | | | | |
|  | Control | | | | | | | | 1µM | | | | | | 10µM | |
|  | 0/3 | | | | | | | | 3/3* | | | | | | 3/3* | |
|  | Average number of pseudonodules per replicate | | | | | | | | | | | | | | | |
|  | 1µM | | | | | | | | | | 10µM | | | | | |
|  | 85.7 | | | | | | | | | | 36.3 | | | | | |
| *Helianthus annuus* | Ratio of replicates producing pseudonodules | | | | | | | | | | | | | | | |
|  | Control | | | | 100nM | | | | | | | | | 20µM | | |
|  | 0/5 | | | | 0/5 | | | | | | | | | 0/5 | | |
|  | Average number of pseudonodules per replicate | | | | | | | | | | | | | | | |
|  | NA | | | | | | | | | | | | | | | |
| *Lotus japonicus* | Ratio of replicates producing pseudonodules | | | | | | | | | | | | | | | |
|  | Control | 10nM | | | | | | 100nM | | | | 1µM | | | | 10µM |
|  | 0/18 | 3/20 | | | | | | 18/19* | | | | 4/20* | | | | 3/20 |
|  | Average number of pseudonodules per replicate | | | | | | | | | | | | | | | |
|  | 10nM | | 100nM | | | | | | | 1µM | | | | | | 10µM |
|  | 0.35 | | 7.9 | | | | | | | 0.3 | | | | | | 0.15 |

**Supplementary Table 2.** ***Cont.***

| *Macroptilium atropurpureum* | Ratio of replicates producing pseudonodules | | | | | | | | | | | | | | | | | | | | | | | | | | |
| --- | --- | --- | --- | --- | --- | --- | --- | --- | --- | --- | --- | --- | --- | --- | --- | --- | --- | --- | --- | --- | --- | --- | --- | --- | --- | --- | --- |
|  | Control | | | | | | | | | | 5µM | | | | | | | | | | | | 10µM | | | | |
|  | 0/10 | | | | | | | | | | 0/10 | | | | | | | | | | | | 9/9* | | | | |
|  | Average number of pseudonodules per replicate | | | | | | | | | | | | | | | | | | | | | | | | | | |
|  | 10µM | | | | | | | | | | | | | | | | | | | | | | | | | | |
|  | 4.8 | | | | | | | | | | | | | | | | | | | | | | | | | | |
| *Medicago sativa* | Ratio of replicates producing pseudonodules | | | | | | | | | | | | | | | | | | | | | | | | | | |
|  | Control | | | 10nM | | | | | | | | | 100nM | | | | | | 1µM | | | | | | | 10µM | |
|  | 8/19 | | | 5/19 | | | | | | | | | 4/20 | | | | | | 10/20* | | | | | | | 14/20* | |
|  | Average number of pseudonodules per replicate | | | | | | | | | | | | | | | | | | | | | | | | | | |
|  | Control | | | 10nM | | | | | | | | | | 100nM | | | | | | | 1µM | | | | | | 10µM |
|  | 0.9 | | | 1 | | | | | | | | | | 0.4 | | | | | | | 0.7 | | | | | | 1.7 |
| *Medicago truncatula* | Ratio of replicates producing pseudonodules | | | | | | | | | | | | | | | | | | | | | | | | | | |
|  | Control | | 10nM | | | | | | | | | 100nM | | | | | | 1µM | | | | | | | 10µM | | |
|  | 0/20 | | 0/20 | | | | | | | | | 2/20 | | | | | | 9/20* | | | | | | | 4/20* | | |
|  | Average number of pseudonodules per replicate | | | | | | | | | | | | | | | | | | | | | | | | | | |
|  | 10nM | | | | 100nM | | | | | | | | | | | 1µM | | | | | | | | | 10µM | | |
|  | 0 | | | | 0.15 | | | | | | | | | | | 0.9 | | | | | | | | | 0.2 | | |
| *Mimosa pudica*  (growing on plates) | Ratio of replicates producing pseudonodules | | | | | | | | | | | | | | | | | | | | | | | | | | |
|  | Control | | 5µM | | | | | | | | | 10µM | | | | | | 15µM | | | | | | | 20µM | | |
|  | 0/10 | | 8/10* | | | | | | | | | 8/10* | | | | | | 8/10* | | | | | | | 1/10 | | |
|  | Average number of pseudonodules per replicate | | | | | | | | | | | | | | | | | | | | | | | | | | |
|  | 5µM | | | | 10µM | | | | | | | | | | | 15µM | | | | | | | | | 20µM | | |
|  | 3.4 | | | | 2.9 | | | | | | | | | | | 2 | | | | | | | | | 0.1 | | |
| *Mimosa pudica*  (growing on vermiculite) | Ratio of replicates producing pseudonodules | | | | | | | | | | | | | | | | | | | | | | | | | | |
|  | Control | | | | 1µM | | | | | | | | | | | 5µM | | | | | | | | | 10µM | | |
|  | 0/3 | | | | 2/3* | | | | | | | | | | | 3/3* | | | | | | | | | 2/3* | | |
|  | Average number of pseudonodules per replicate | | | | | | | | | | | | | | | | | | | | | | | | | | |
|  | 1µM | | | | | | 5µM | | | | | | | | | | | | | | | 10µM | | | | | |
|  | 1.3 | | | | | | 7.7 | | | | | | | | | | | | | | | 2.3 | | | | | |
| *Nicotiana tabacum* | Ratio of replicates producing pseudonodules | | | | | | | | | | | | | | | | | | | | | | | | | | |
|  | Control | | | | | | | 1µM | | | | | | | | | | | | 10µM | | | | | | | |
|  | 0/10 | | | | | | | 0/10 | | | | | | | | | | | | 0/10 | | | | | | | |
|  | Average number of pseudonodules per replicate | | | | | | | | | | | | | | | | | | | | | | | | | | |
|  | NA | | | | | | | | | | | | | | | | | | | | | | | | | | |
| *Pisum sativum* | Ratio of replicates producing pseudonodules | | | | | | | | | | | | | | | | | | | | | | | | | | |
|  | Control | | | | | | | | 1µM | | | | | | | | 5µM | | | | | | | | 10µM | | |
|  | 0/3 | | | | | | | | N/A† | | | | | | | | N/A† | | | | | | | | N/A† | | |
|  | Average number of pseudonodules per replicate | | | | | | | | | | | | | | | | | | | | | | | | | | |
|  |  | | | | | | | | | | N/A | | | | | | | | | | | | | | | | |
| *Sesbania rostrata* | | Ratio of replicates producing pseudonodules | | | | | | | | | | | | | | | | | | | | | | | | | |
|  |  | Control | | | | 1µM | | | | | | | | | 5µM | | | | | | | | | 10µM | | | |
|  |  | 0/3 | | | | 3/3* | | | | | | | | | 3/3* | | | | | | | | | 3/3* | | | |
|  |  | Average number of pseudonodules per replicate | | | | | | | | | | | | | | | | | | | | | | | | | |
|  |  | 1µM | | | | | | | | 5µM | | | | | | | | | | | | 10µM | | | | | |
|  |  | 17 | | | | | | | | 5 | | | | | | | | | | | | 7.3 | | | | | |

**Supplementary Table 2.** ***Cont.*** †: *Pisum sativum* pseudonodules could not be enumerated on

account of the significant root swelling that was difficult to distinguish from pseudonodules without sectioning.

| *Solanum lycopersicum* | Ratio of replicates producing pseudonodules | | | | | | | | | | | | |
| --- | --- | --- | --- | --- | --- | --- | --- | --- | --- | --- | --- | --- | --- |
|  | Control | 10µM | | | | 20µM | | | 30µM | | | 40µM | |
|  | 0/10 | 0/10 | | | | 0/10 | | | 0/10 | | | 0/10 | |
|  | Average number of pseudonodules per replicate | | | | | | | | | | | | |
|  | N/A | | | | | | | | | | | | |
| *Trema tomentosa* | Ratio of replicates producing pseudonodules | | | | | | | | | | | | |
|  | Control | | | | 1µM | | | | | | 10µM | | |
|  | 0/10 | | | | 0/10 | | | | | | 0/10 | | |
|  | Average number of pseudonodules per replicate | | | | | | | | | | | | |
|  | NA | | | | | | | | | | | | |
| *Trifolium repens* | Ratio of replicates producing pseudonodules | | | | | | | | | | | | |
|  | Control | | 10nM | | | | 100nM | | | 1µM | | | 10µM |
|  | 0/20 | | 3/20 | | | | 4/20* | | | 3/20 | | | 14/20* |
|  | Average number of pseudonodules per replicate | | | | | | | | | | | | |
|  | 10nM | | | 100nM | | | | 1µM | | | | | 10µM |
|  | 0.2 | | | 0.6 | | | | 0.4 | | | | | 1.2 |
| *Trifolium subterraneum* | Ratio of replicates producing pseudonodules | | | | | | | | | | | | |
|  | Control | | 5µM | | | | 10µM | | | 15µM | | | 20µM |
|  | 0/5 | | 9/10* | | | | 8/10* | | | 4/10 | | | 9/10* |
|  | Average number of pseudonodules per replicate | | | | | | | | | | | | |
|  | 5µM | | | 10µM | | | | 15µM | | | | | 20µM |
|  | 4.4 | | | 2.5 | | | | 1.1 | | | | | 2.7 |
| *Zea mays* | Ratio of replicates producing pseudonodules | | | | | | | | | | | | |
|  | Control | | | | 100nM | | | | | | 20µM | | |
|  | 0/10 | | | | 0/10 | | | | | | 0/10 | | |
|  | Average number of pseudonodules per replicate | | | | | | | | | | | | |
|  | NA | | | | | | | | | | | | |

**Supplementary Table 2.** ***Cont.***
